# Supplementary material for: Simplified synthetic routes for low cost and high photovoltaic performance n-type organic semiconductor acceptors
Source: Nat Commun. 2019 Jan 31;10:519. doi: 10.1038/s41467-019-08508-3 (PMC6355909; doi:10.1038/s41467-019-08508-3)
Supplement: Supplementary file 1 — Supplementary Information [file 41467_2019_8508_MOESM1_ESM.pdf]

Simplified synthetic routes for low cost and high photovoltaic performance *n*-type organic semiconductor acceptors

Xiaojun Li,[a b] Fei Pan,[a b] Chenkai Sun,[a b] Ming Zhang[c], Zhiwei Wang,[d e] Jiaqi Du,[a b] Jing Wang[c], Min Xiao,[d e] Lingwei Xue,[a] Zhi-Guo Zhang,[a] Chunfeng Zhang,[d e]* Feng Liu[c], *Yongfang Li[a b f]*

[a] Beijing National Laboratory for Molecular Sciences, CAS Key Laboratory of Organic Solids, Institute of Chemistry, Chinese Academy of Sciences, Beijing 100190, China;

[b] School of Chemical Science, University of Chinese Academy of Sciences, Beijing 100049, China;

[c] Department of Physics and Astronomy and Collaborative Innovation Center of IFSA (CICIFSA) Shanghai Jiaotong University, Shanghai 200240, China;

[d] National Laboratory of Solid State Microstructures, School of Physics, and Collaborative Innovation Center of Advanced Microstructures, Nanjing University, Nanjing 210093, China;

[e] Synergetic Innovation Center in Quantum Information and Quantum Physics, University of Science and Technology of China, Hefei, Anhui 230026, China.

[f] Laboratory of Advanced Optoelectronic Materials, College of Chemistry, Chemical Engineering and Materials Science, Soochow University, Suzhou, Jiangsu 215123, China;

Supplementary Figures

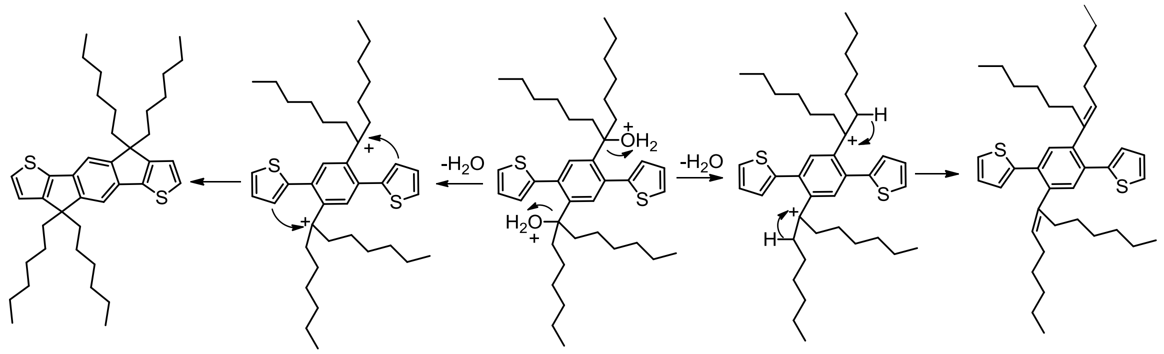

Supplementary Figure 1. Ring closure *vs* alkene formation

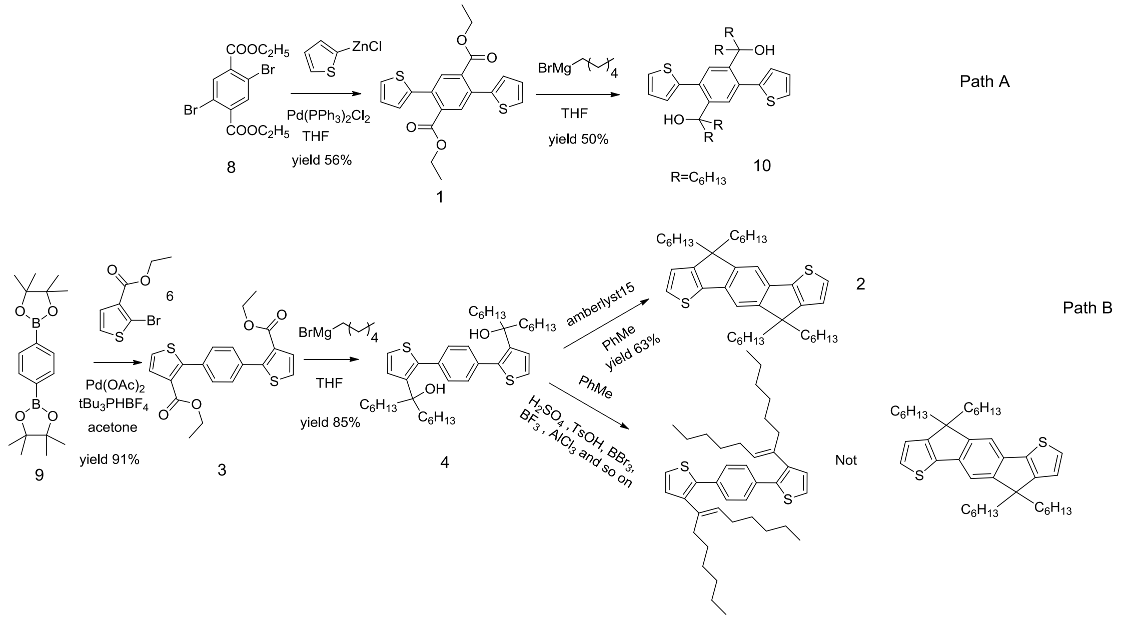

Supplementary Figure 2. Selection of synthetic paths

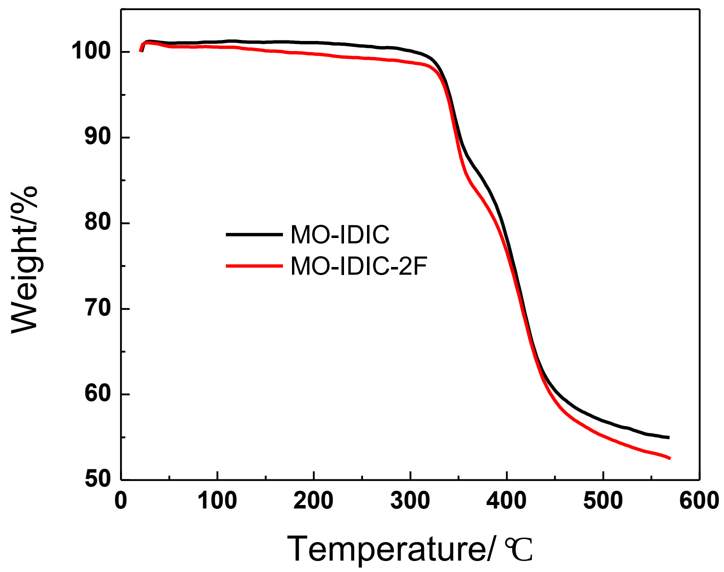

Supplementary Figure 3. TGA plots of MO-IDIC and MO-IDIC-2F

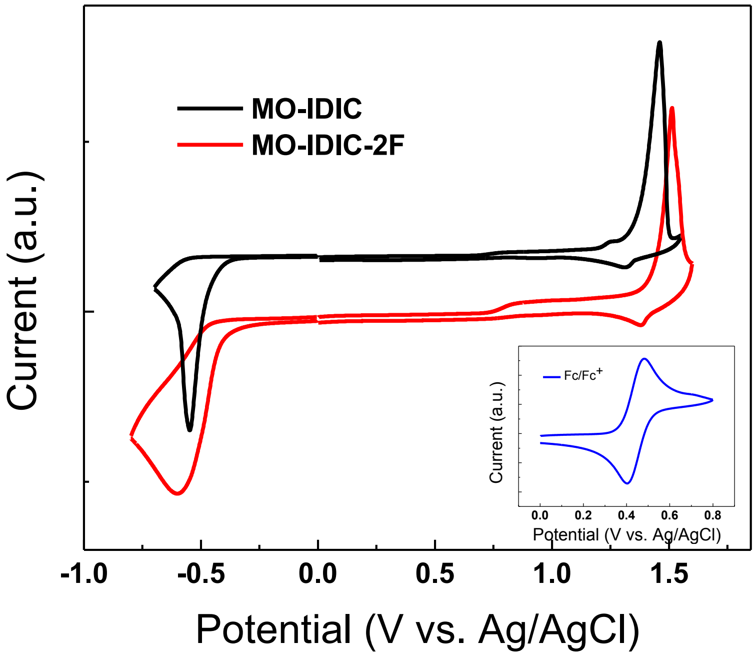

Supplementary Figure 4. Cyclic voltammograms of MO-IDIC and MO-IDIC-2F, the inset shows the cyclic voltammogram of ferrocene/ferrocenium (Fc/Fc^{+}) couple used as an internal reference

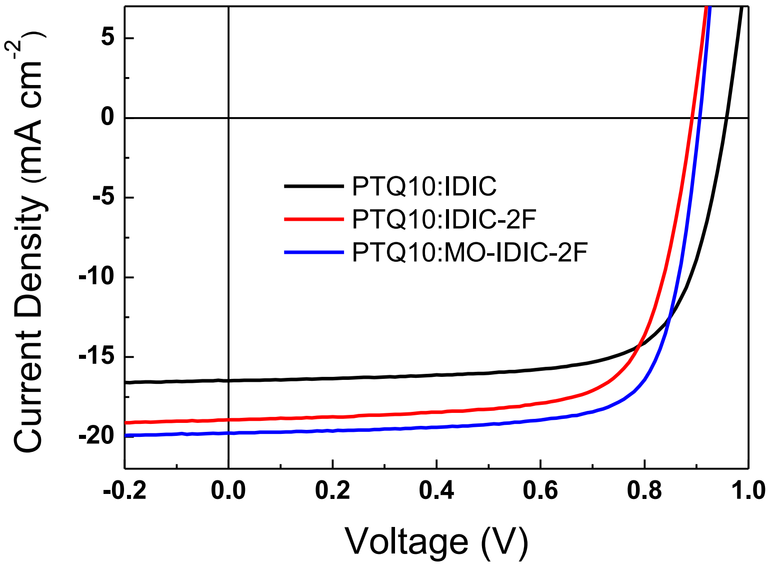

Supplementary Figure 5. *J-V* curves of the optimized PSCs based on PTQ10: acceptors (1:1) with thermal annealing at 140 ^{o}C (for the IDIC-based devices) and 120 ^{o}C (for the IDIC-2F-based devices) or 110 ^{o}C (for the MO-IDIC-2F-based devices) for 5 min, under the illumination of AM 1.5G, 100 mW cm^{-2}

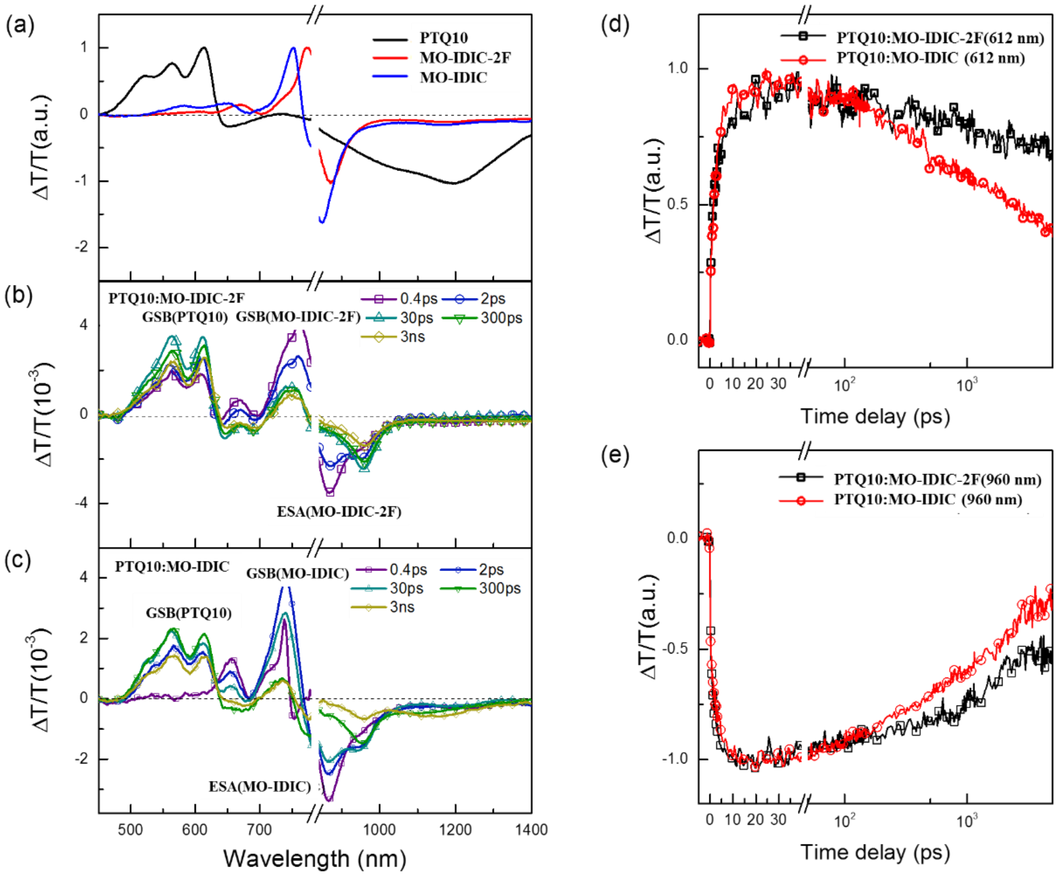

Supplementary Figure 6. **Transient absorption properties of the photovoltaic materials and active layers of the PSCs**: (a) Transient absorption (TA) spectra recorded from neat films of PTQ10, MO-IDIC-2F and MO-IDIC at delay time of 1ps. (b) TA spectra recorded from the PTQ10/MO-IDIC-2F blend film at different time delays. (c) TA spectra recorded from the PTQ10/MO-IDIC blend film at different time delays. (d) The kinetic curves probed at 612 nm in the blend films of PTQ10/MO-IDIC and PTQ10/MO-IDIC-2F. (e) The kinetic traces probed at 960 nm in the systems of PTQ10/MO-IDIC and PTQ10/MO-IDIC-2F.

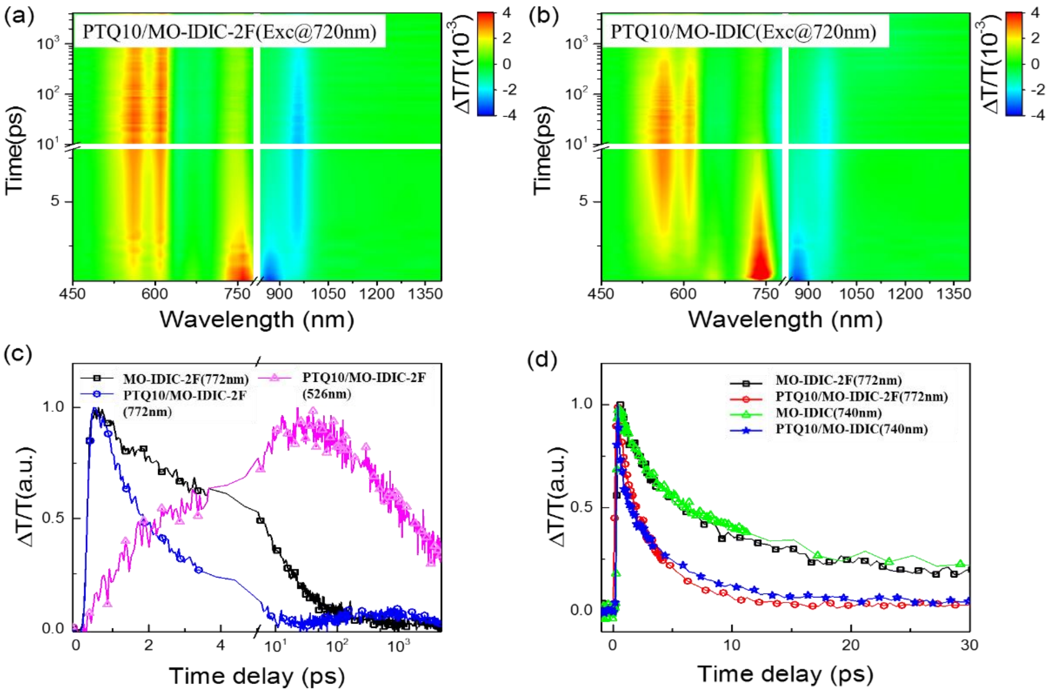

Supplementary Figure 7. Fs-resolved transient absorption (TA) experiments were performed on (a) PTQ10/MO-IDIC-2F and (b) PTQ10/MO-IDIC blend films with pump wavelength at 720 nm. Kinetic curves recorded at 772nm in neat film of MO-IDIC-2F and probed at 772nm and 526nm in blend film of PTQ10/MO-IDIC. (c) The early-stage decay of GSB at 772 nm (GSB of MO-IDIC) is dramatically shortened in blend, while the GSB at 612 nm (GSB of PTQ10) simultaneously builds up. (d) The kinetic traces probed at 772 nm in neat film of MO-IDIC-2F and the blend film of PTQ10/MO-IDIC-2F, which are compared with the decay curves probed at 740 nm in neat film of MO-IDIC and the blend film of PTQ10/MO-IDIC. The difference between the GSB signal of acceptor in the neat films and its blend film is almost same for two systems, implying similar hole transfer rate.

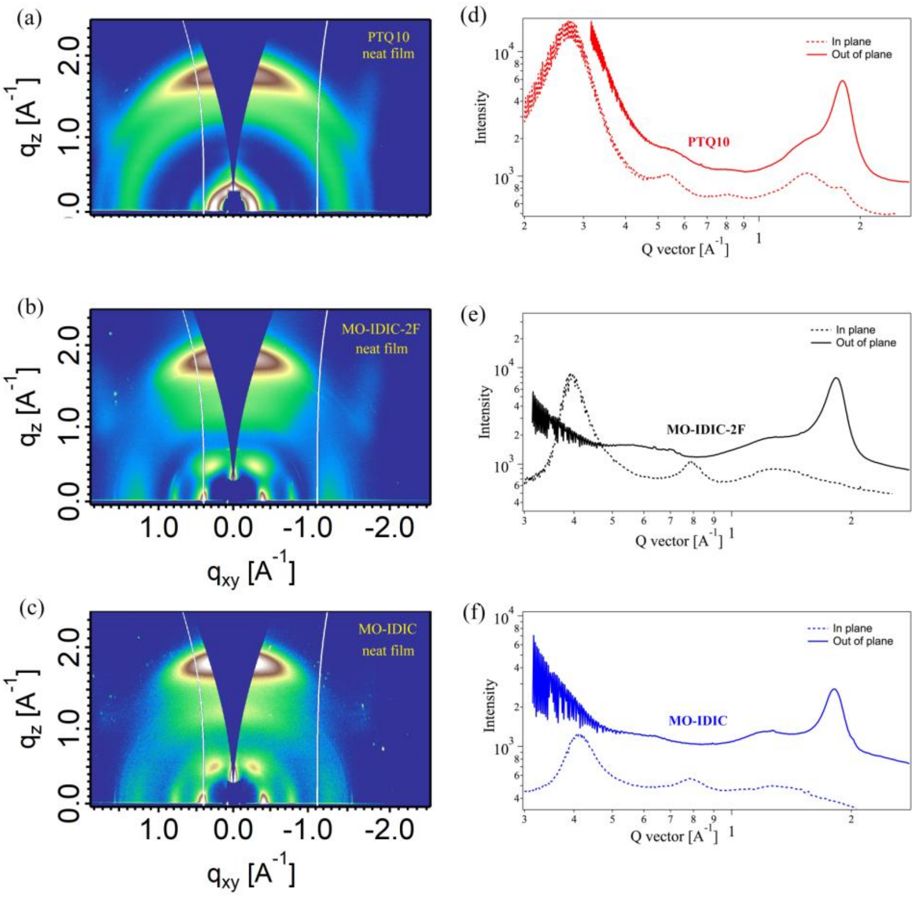

Supplementary Figure 8. 2D GIWAXS patterns of (a) PTQ10 neat film, (b)MO-IDIC-2F neat film, (c) MO-IDIC neat film, Line cuts of the GIWAXS images of (d) PTQ10 neat film, (e)MO-IDIC-2F neat film, (f) MO-IDIC neat film.

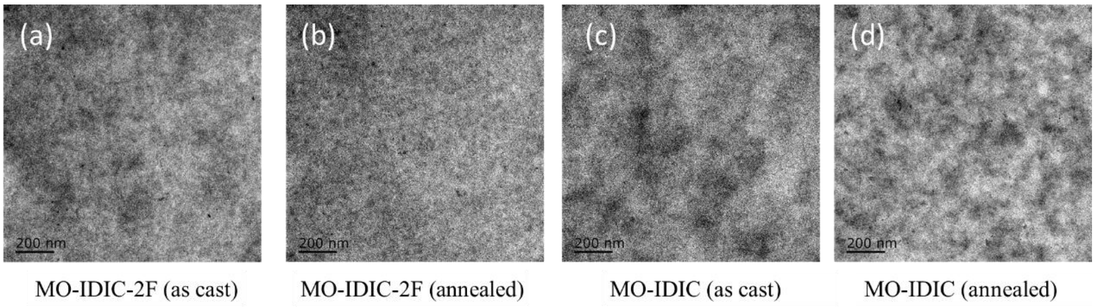

Supplementary Figure 9. TEM images for the blend films of PTQ10 and the acceptors: (a, c) PTQ10:MO-IDIC-2F or MO-IDIC without extra treatment; (b, d) PTQ10:MO-IDIC-2F or MO-IDIC with thermal annealing condition

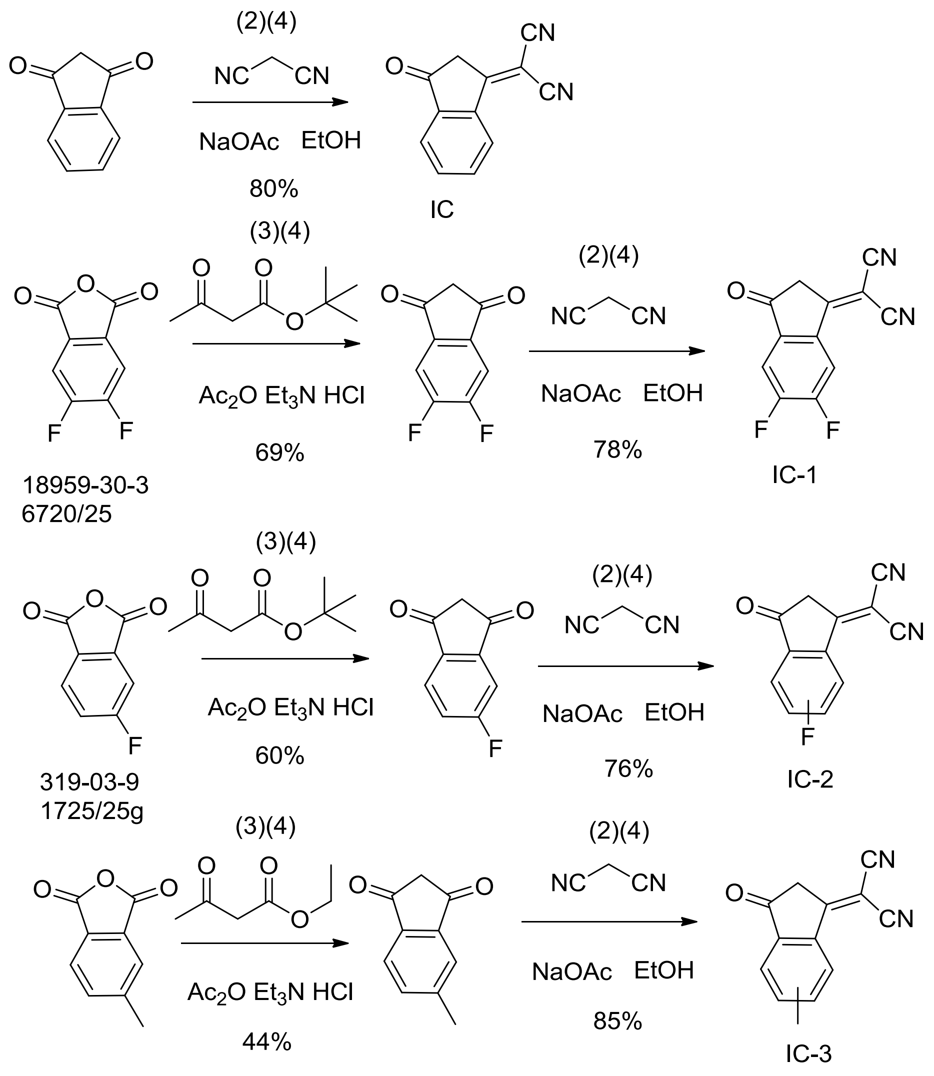

Supplementary Figure 10. Synthetic route of IC, IC-1, IC-2 and IC-3

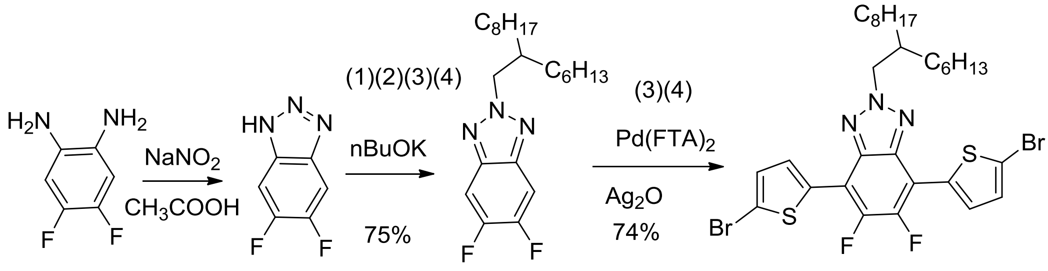

Supplementary Figure 11. Synthetic route of 4,7-bis(5-bromothiophen-2-yl)-5,6 difluoro-2(2hexyldecyl)-2H benzo[d] [1,2,3]triazole

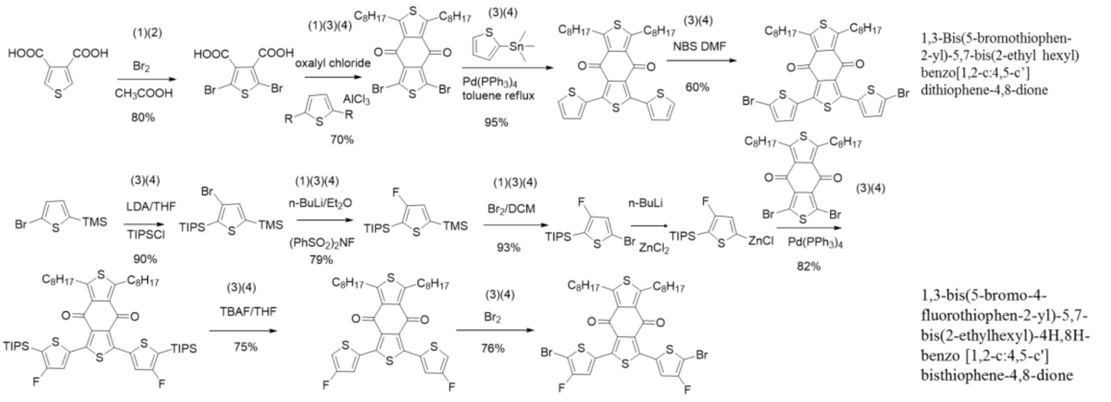

Supplementary Figure 12. Synthetic route of 1,3-Bis(5-bromothiophen-2-yl) -5,7-bis (2-ethyl hexyl) benzo[1,2-c:4,5-c’] dithiophene-4,8-dione and 1,3-bis (5-bromo -4-fluorothiophen-2-yl)-5,7-bis(2-ethylhexyl)-4H,8H-benzo[1,2-c:4,5-c']bisthiophene -4,8-dione

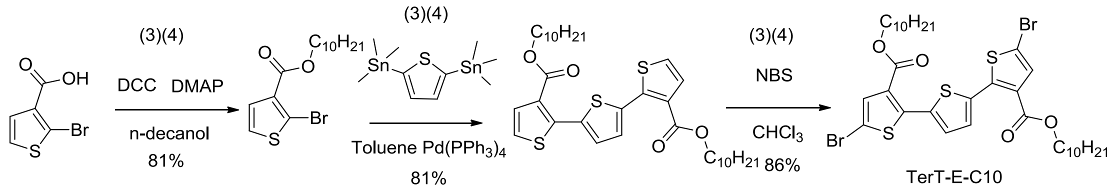

Supplementary Figure 13. Synthetic route of TerT-E-C10

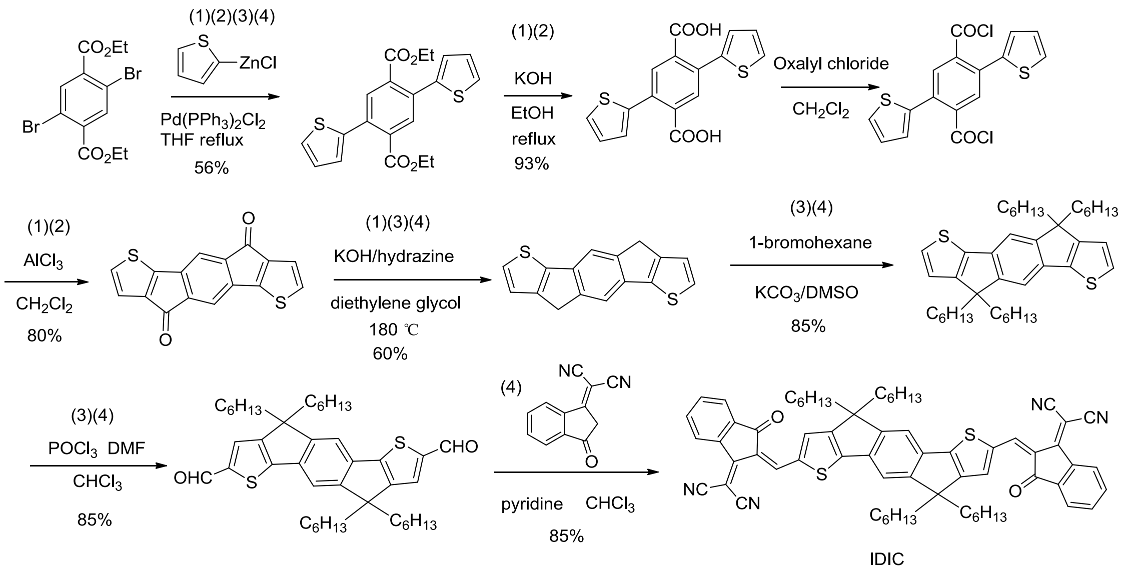

Supplementary Figure 14. Synthetic route of IDIC

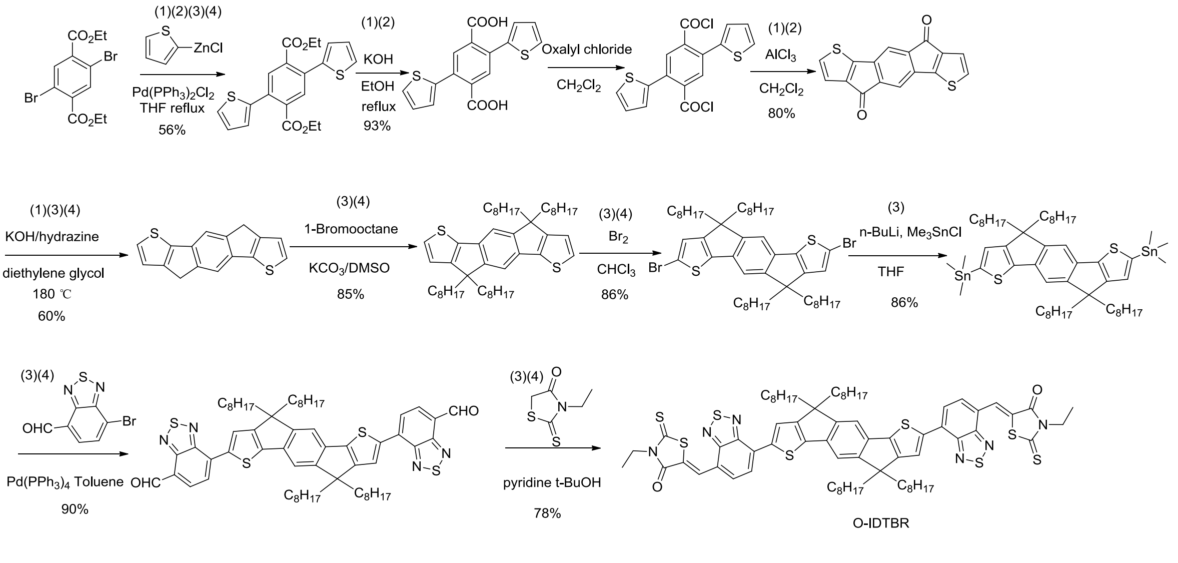

Supplementary Figure 15. Synthetic route of O-IDTBR

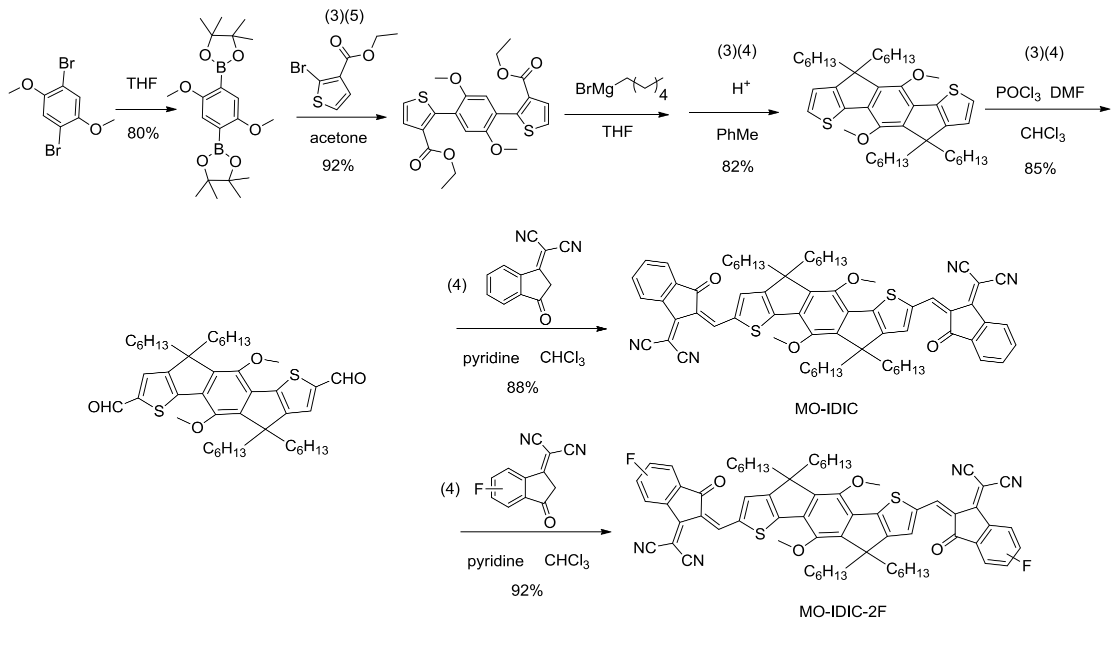

Supplementary Figure 16. Synthetic route of MO-IDIC and MO-IDIC-2F

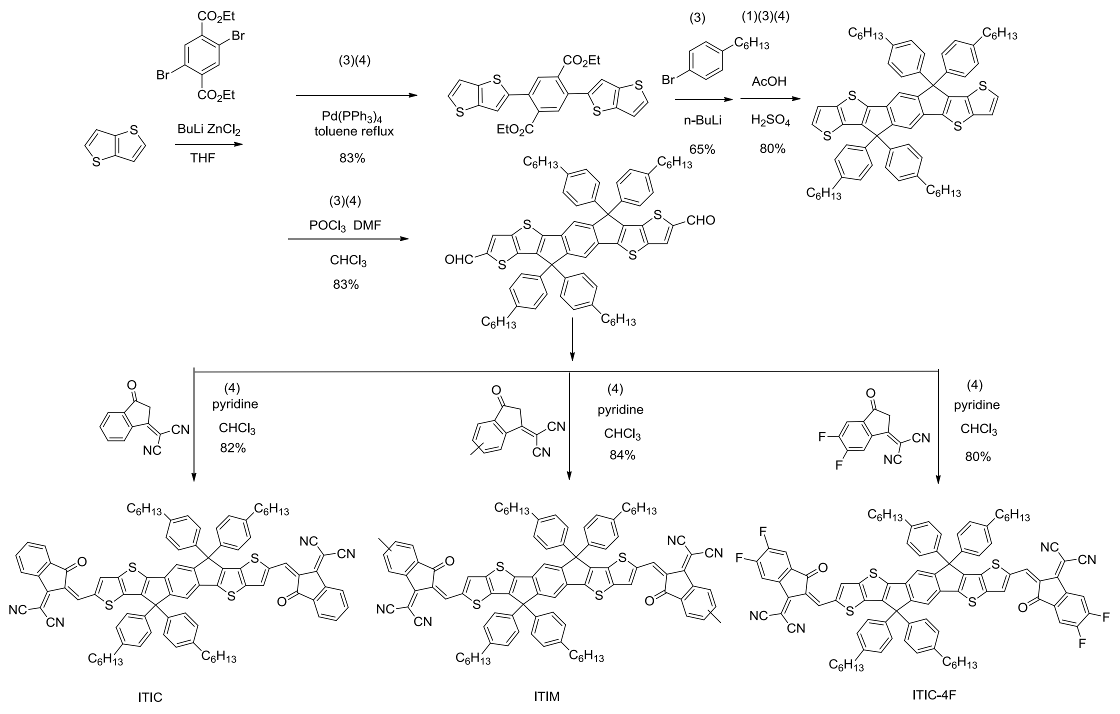

Supplementary Figure 17. Synthetic route of ITIC, ITIM and ITIC-4F

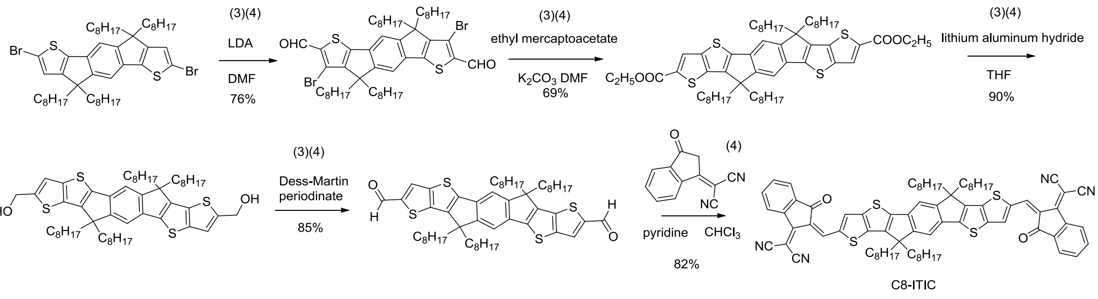

Supplementary Figure 18. Synthetic route of C8-ITIC

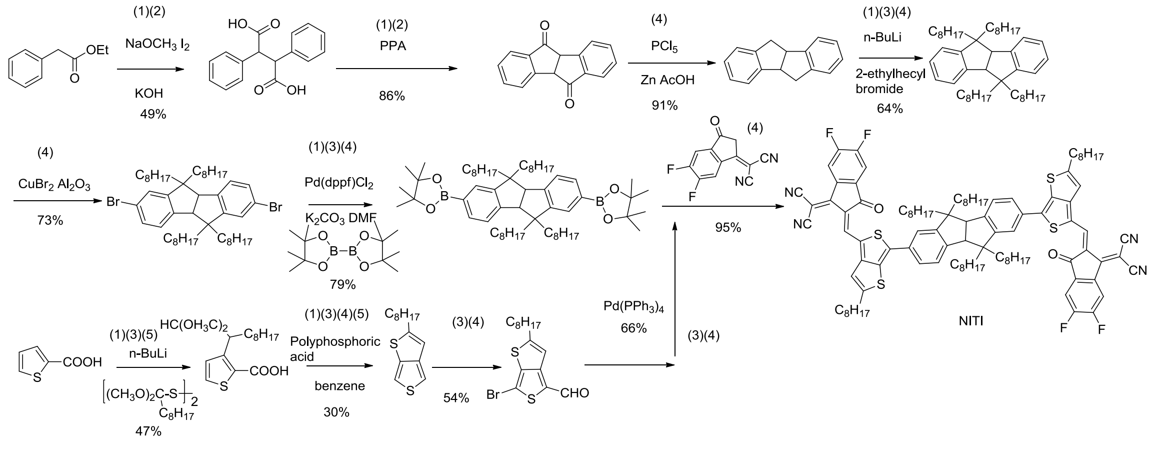

Supplementary Figure 19. Synthetic route of NITI

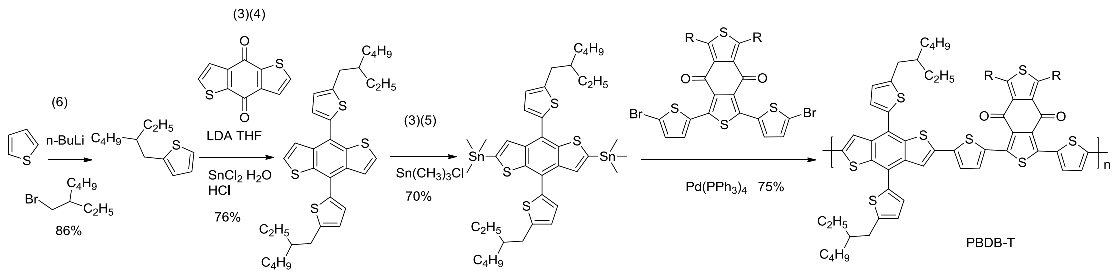

Supplementary Figure 20. Synthetic route of PBDB-T

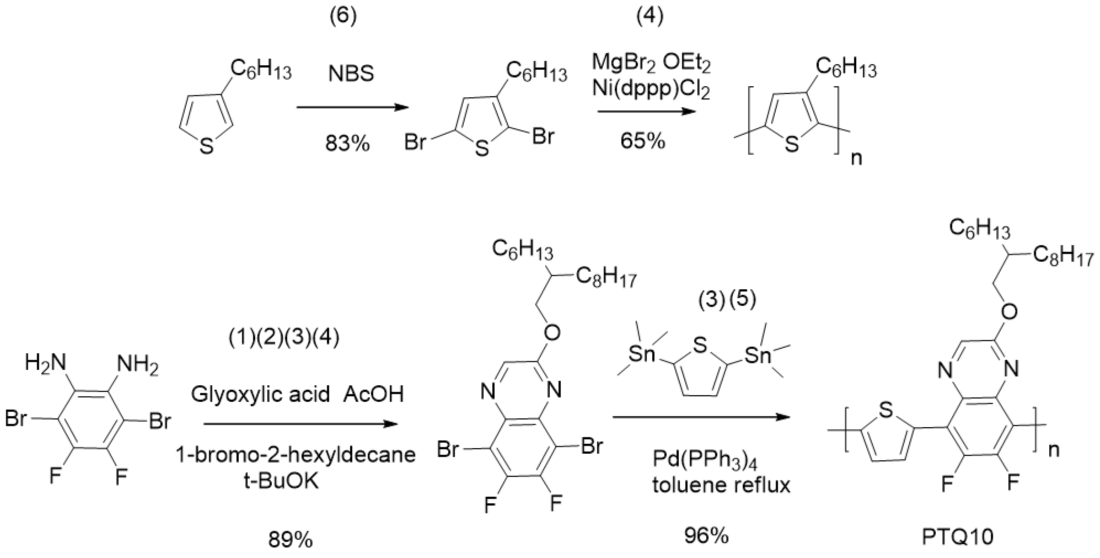

Supplementary Figure 21. Synthetic route of P3HT and PTQ10

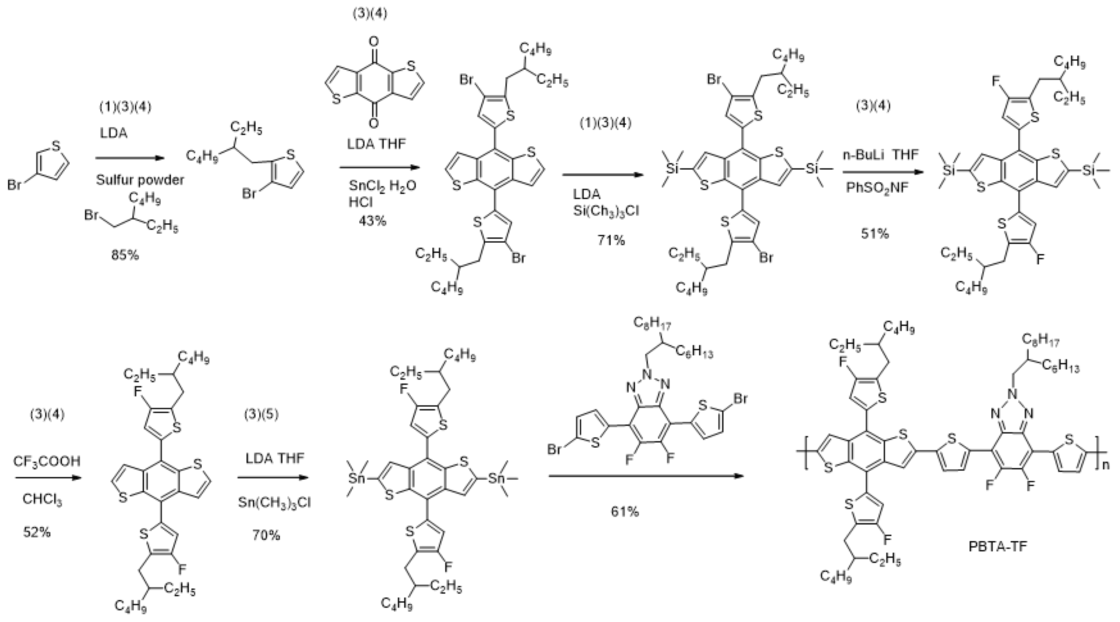

Supplementary Figure 22. Synthetic route of PBTA-TF

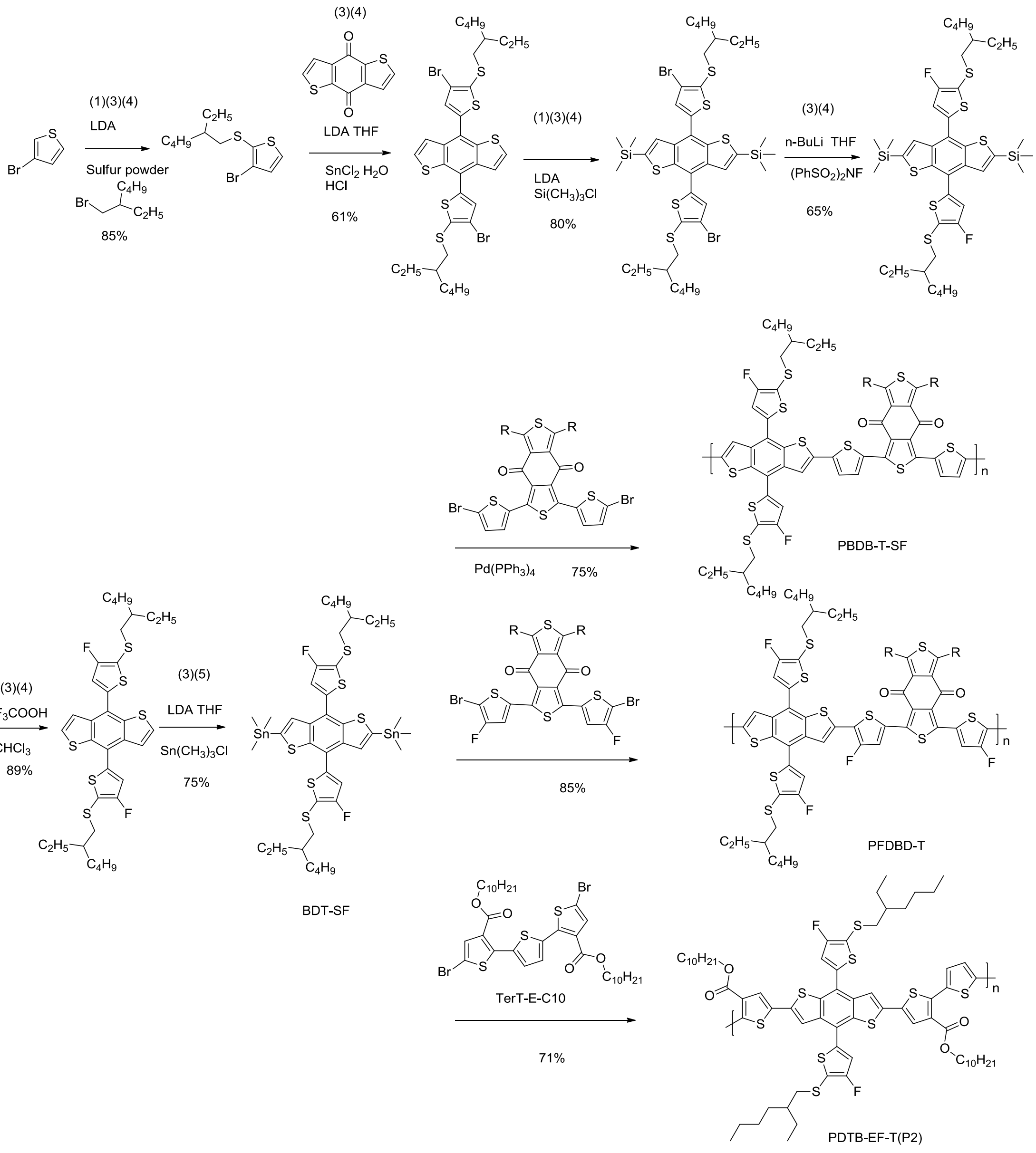

Supplementary Figure 23. Synthetic route of PBDB-T-SF, PFDBD-T and PDTB-EF-T(P2)

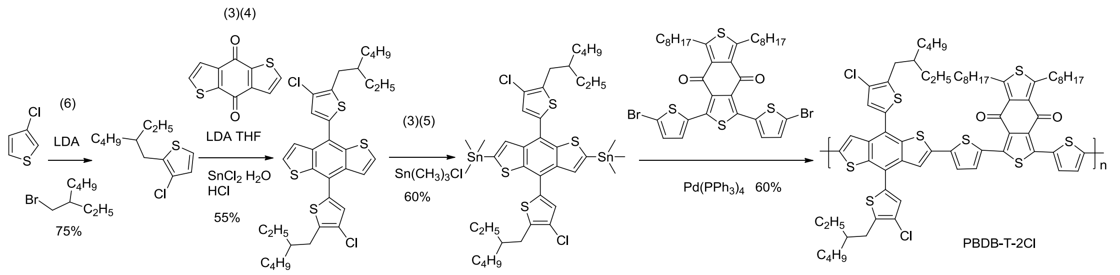

Supplementary Figure 24. Synthetic route of PBDB-T-2Cl

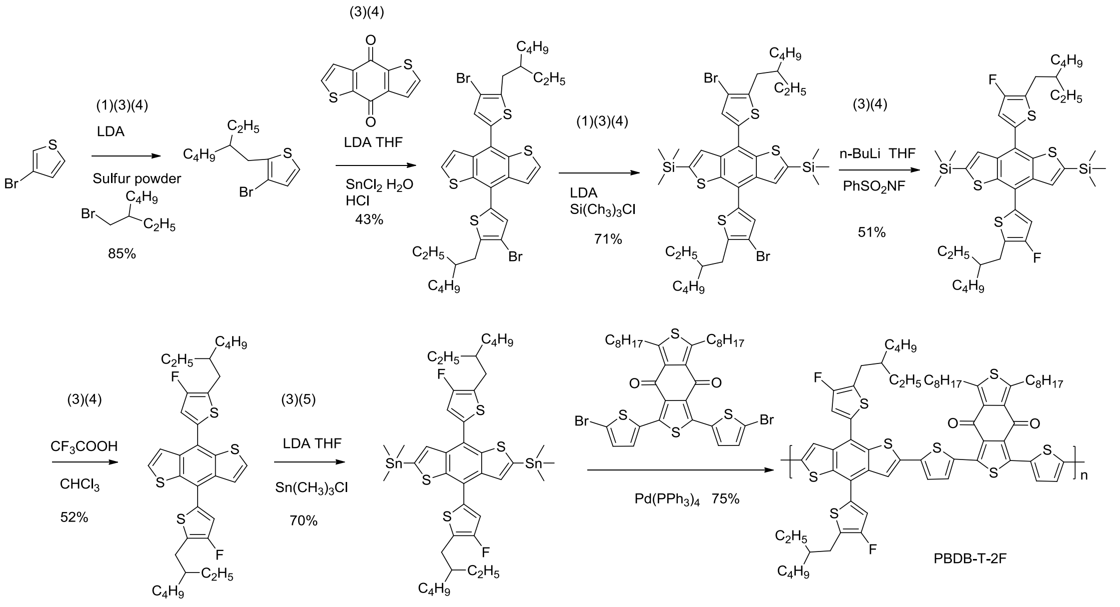

Supplementary Figure 25. Synthetic route of PBDB-T-2F

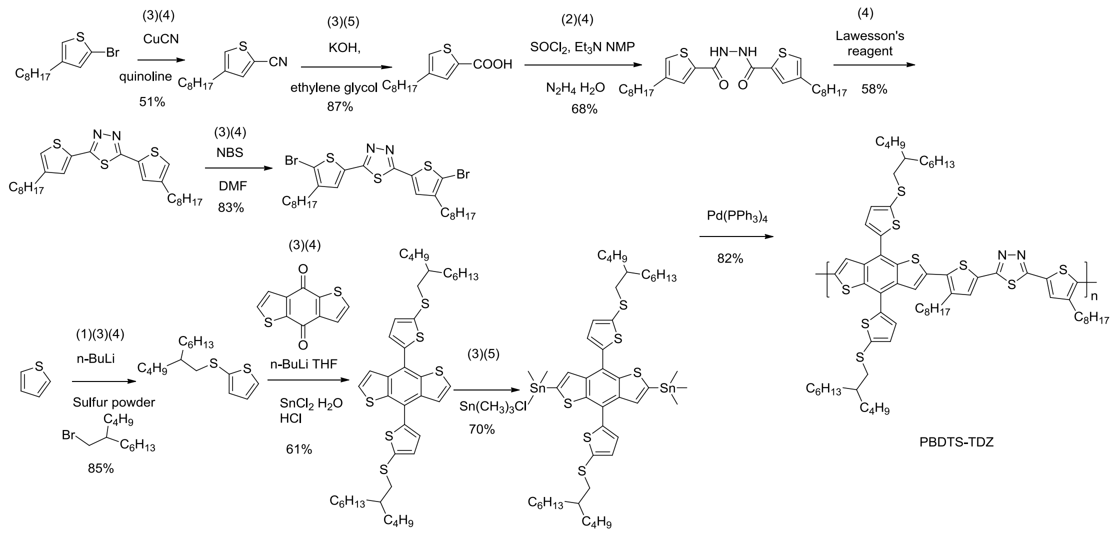

Supplementary Figure 26. Synthetic route of PBDTS-TDZ

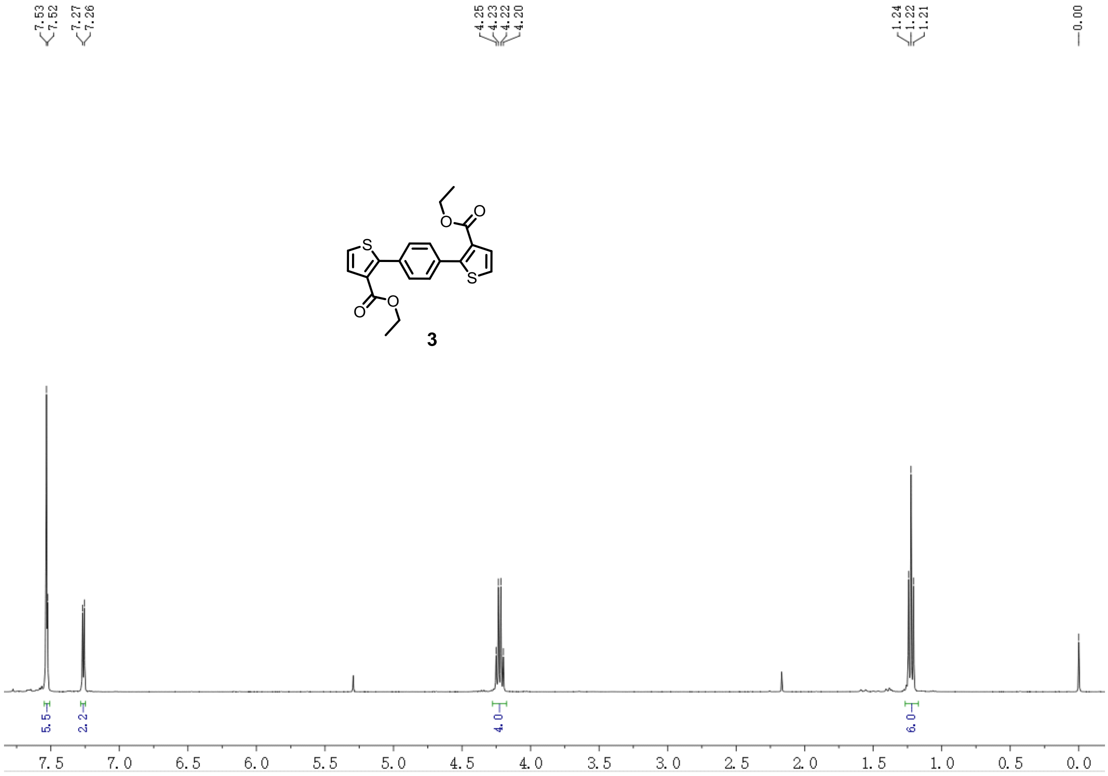

Supplementary Figure 27. ^{1}H NMR spectrum of 3

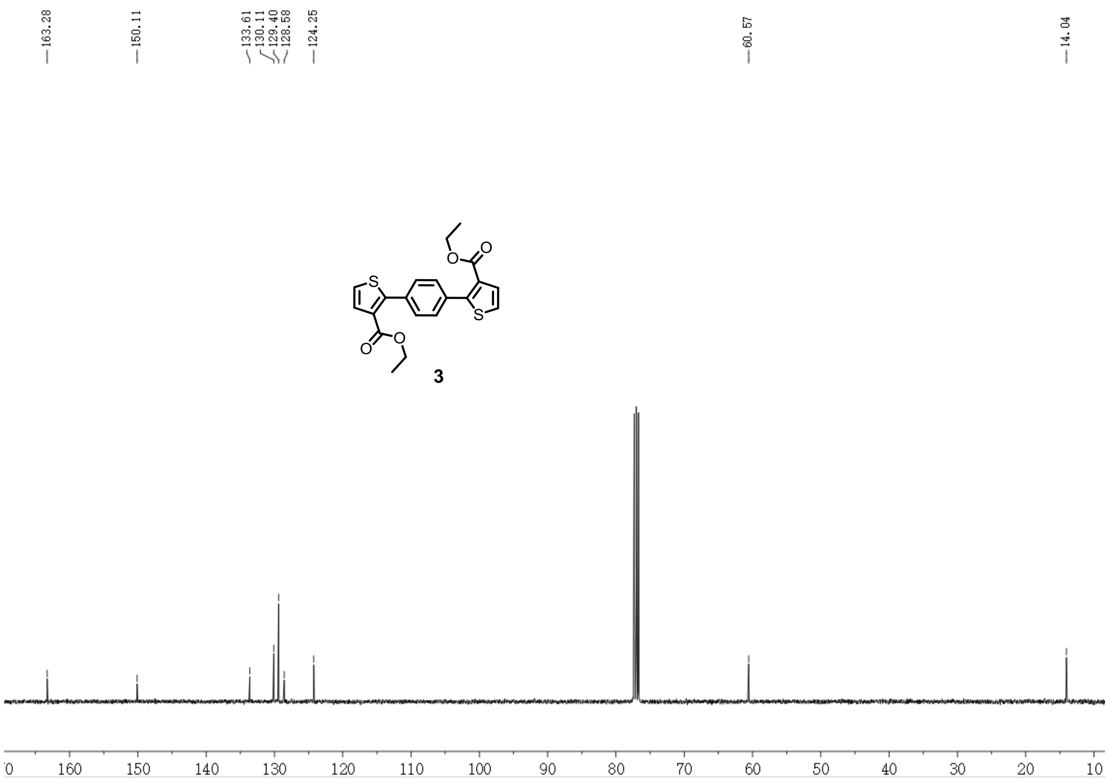

Supplementary Figure 28.^{13}C NMR spectrum of 3

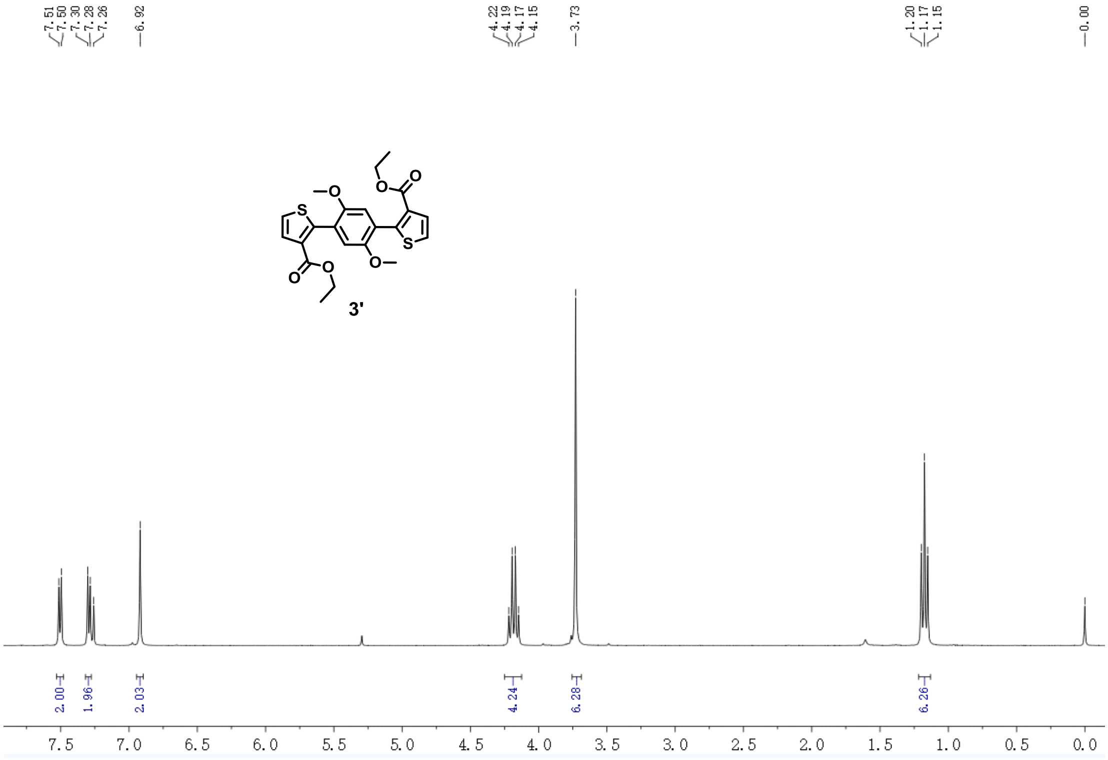

Supplementary Figure 29. ^{1}H NMR spectrum of 3’

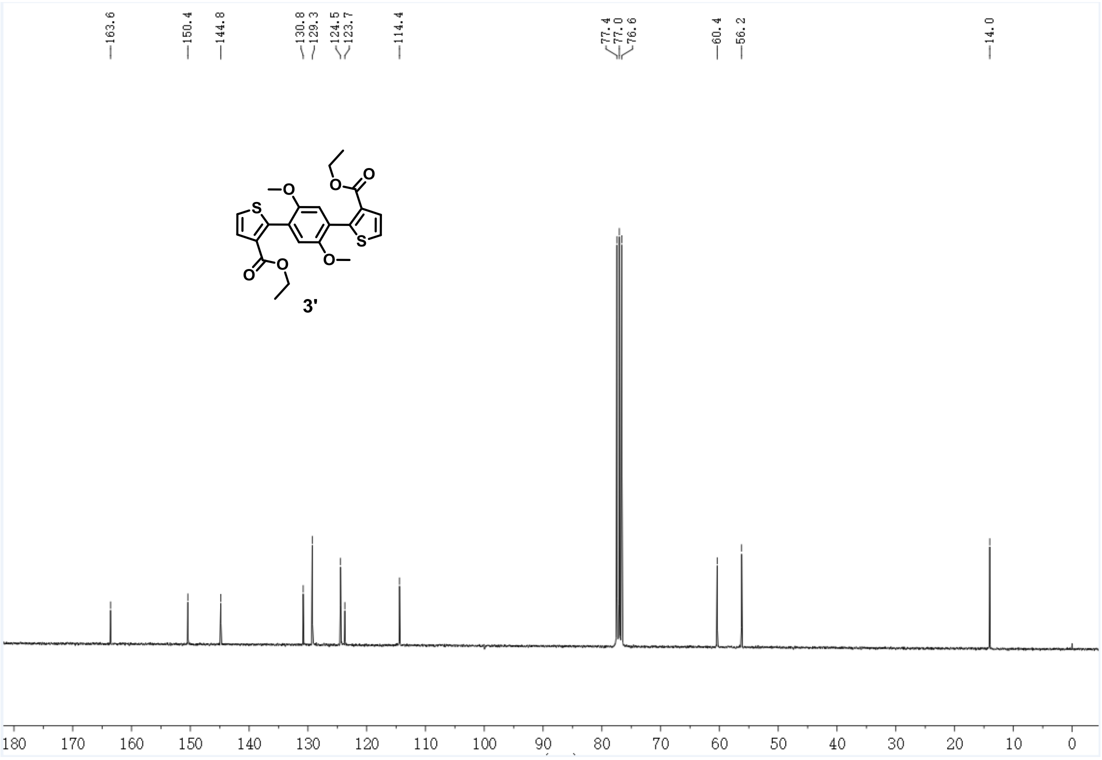

Supplementary Figure 30. ^{13}C NMR spectrum of 3’

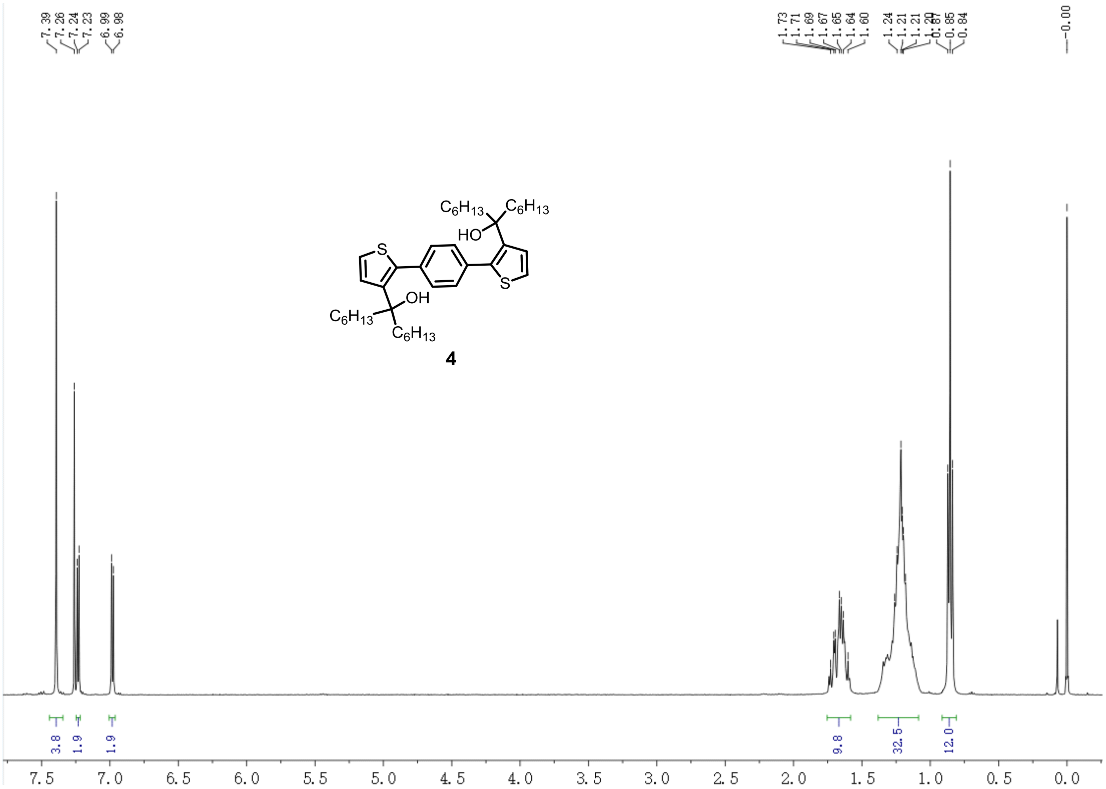

Supplementary Figure 31. ^{1}H NMR spectrum of 4

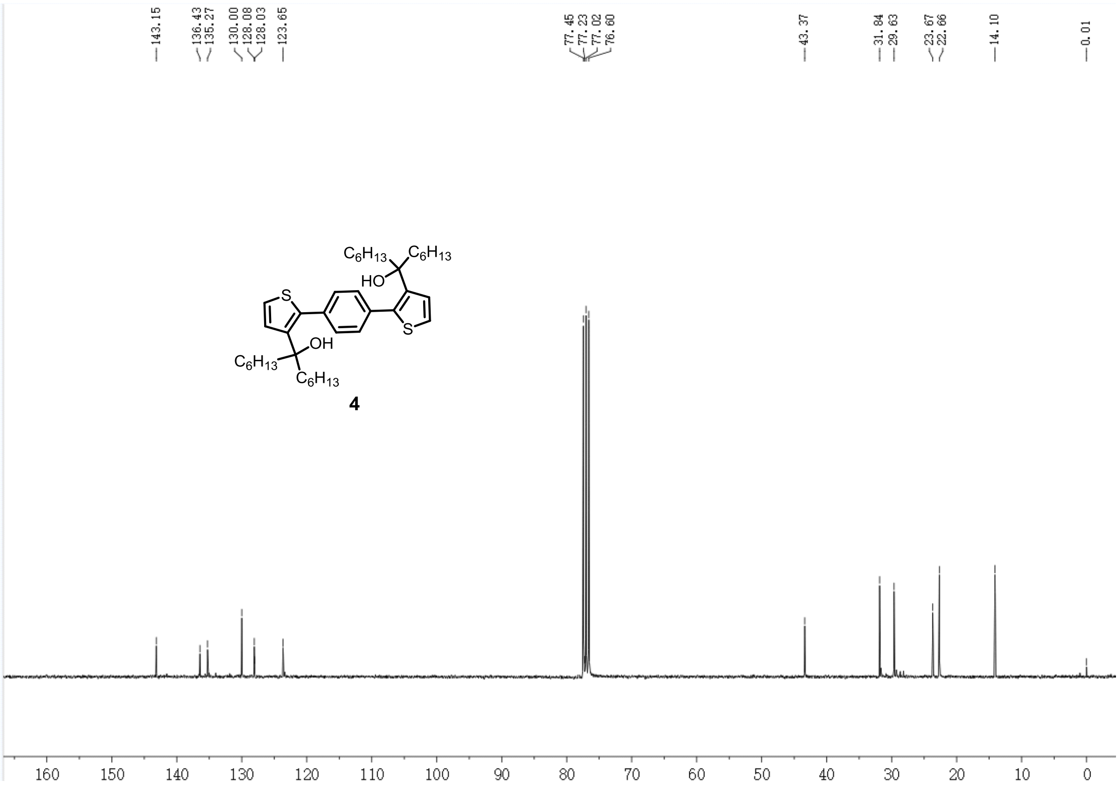

Supplementary Figure 32. ^{13}C NMR spectrum of 4

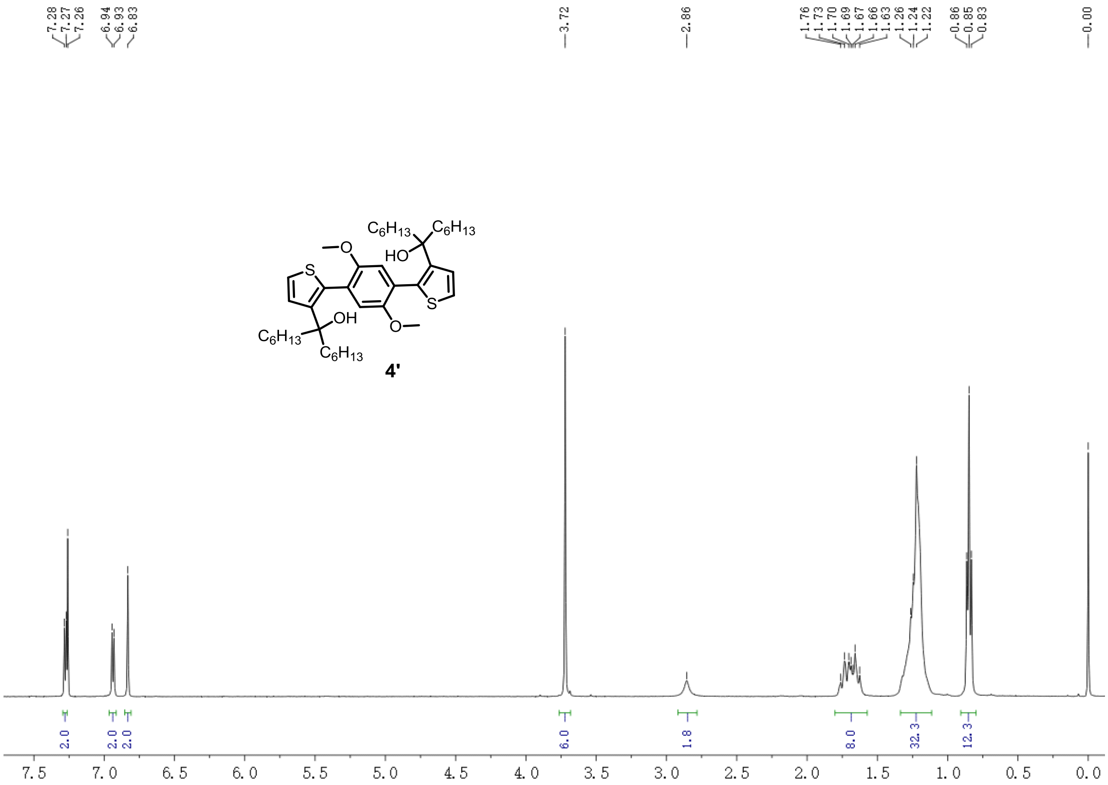

Supplementary Figure 33. ^{1}H NMR spectrum of 4’

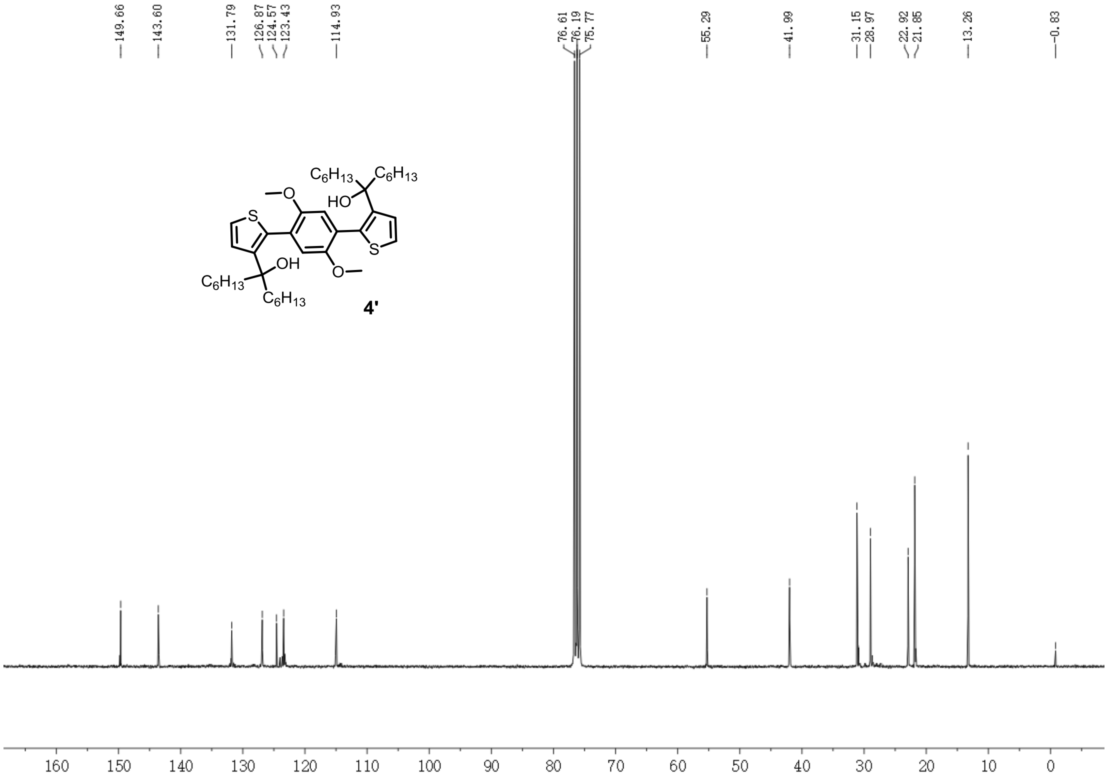

Supplementary Figure 34. ^{13}C NMR spectrum of 4’

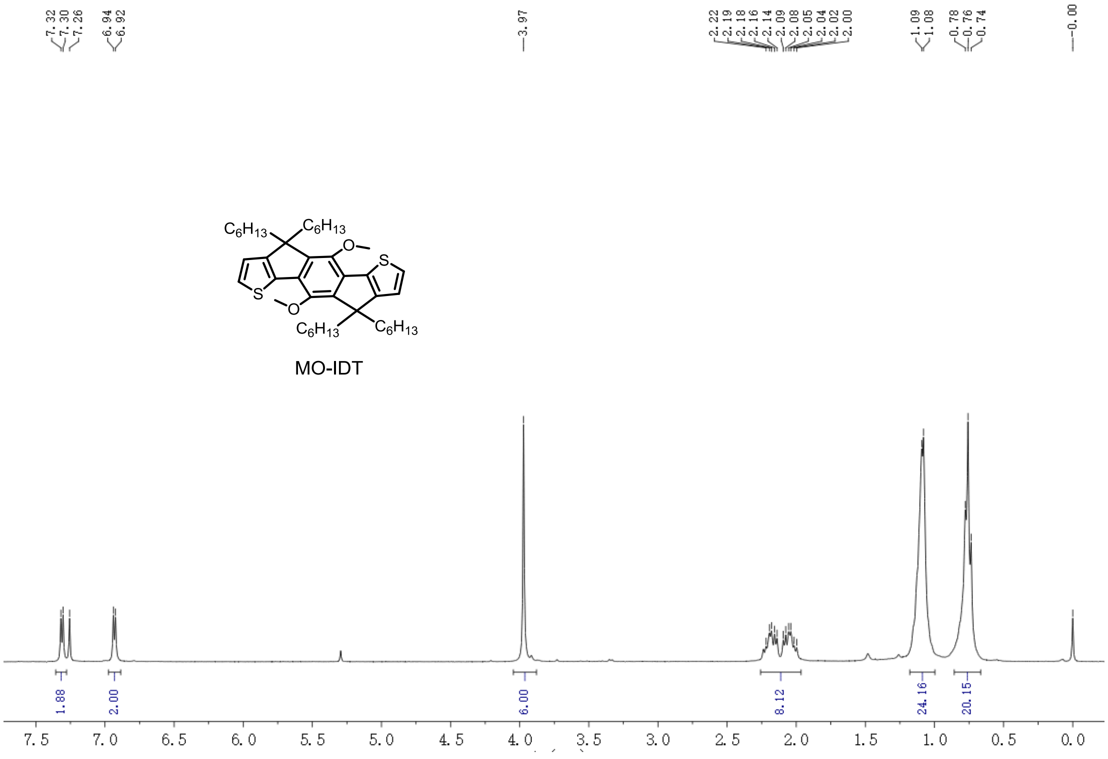

Supplementary Figure 35. ^{1}H NMR spectrum of MO-IDT

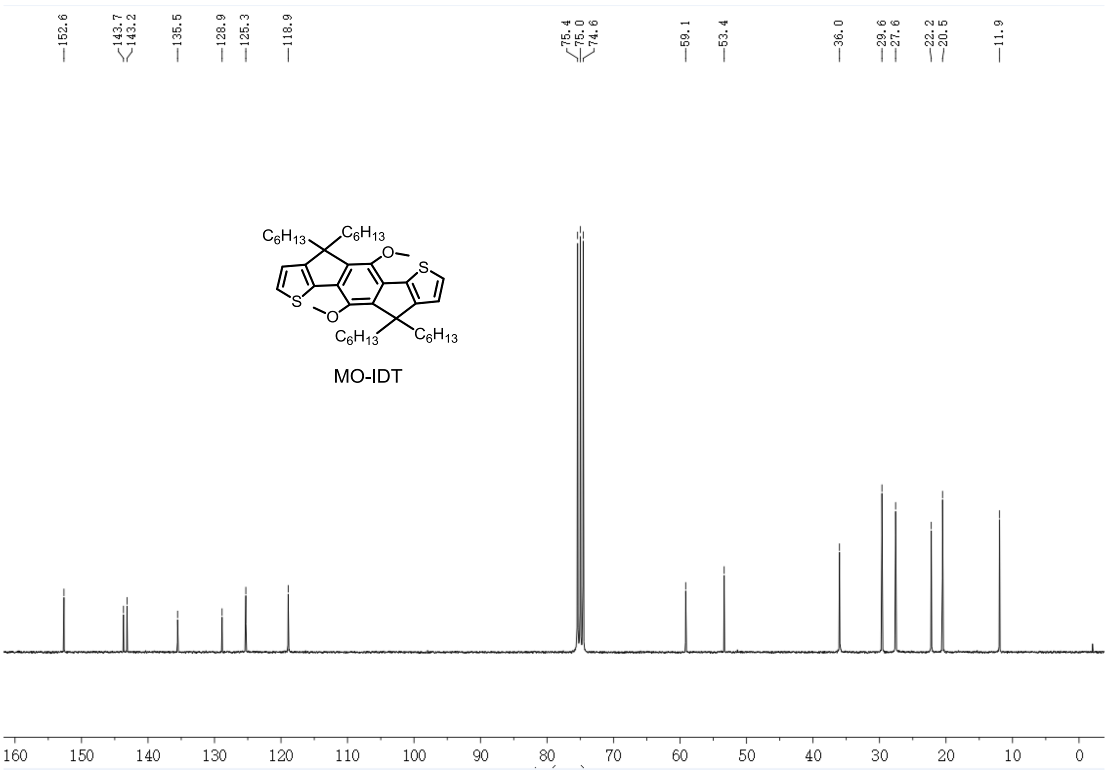

Supplementary Figure 36. ^{13}C NMR spectrum of MO-IDT

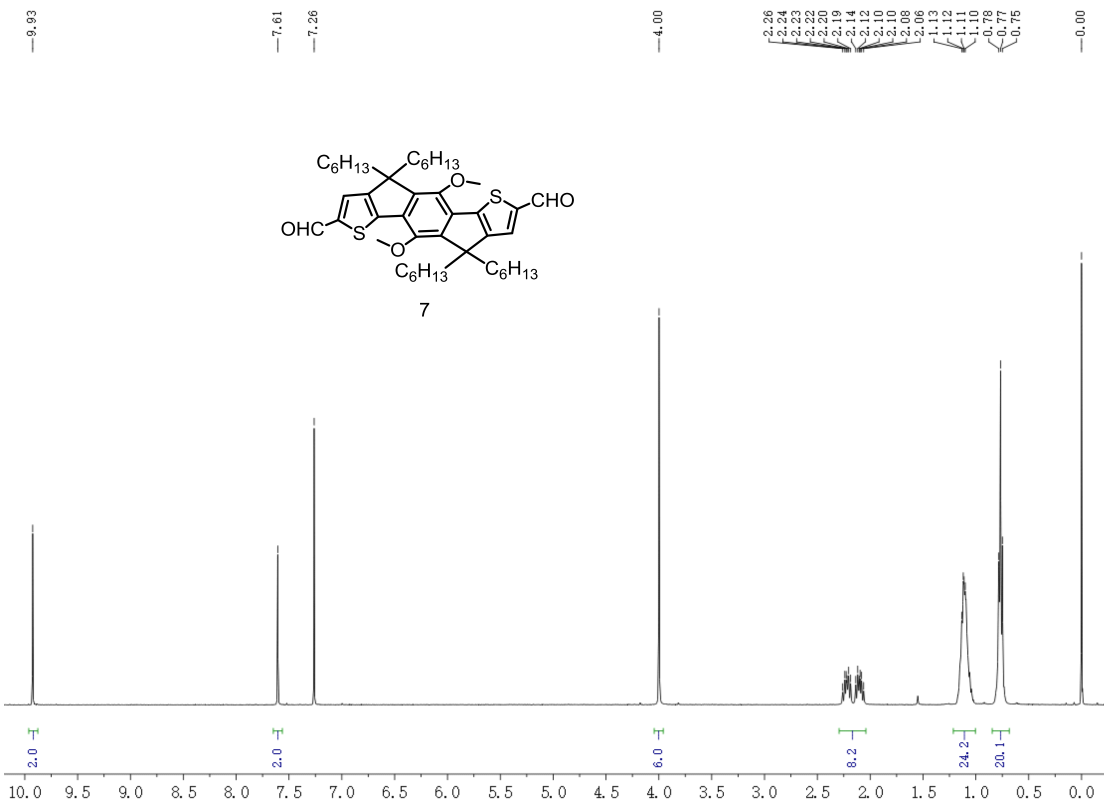

Supplementary Figure 37. ^{1}H NMR spectrum of 7

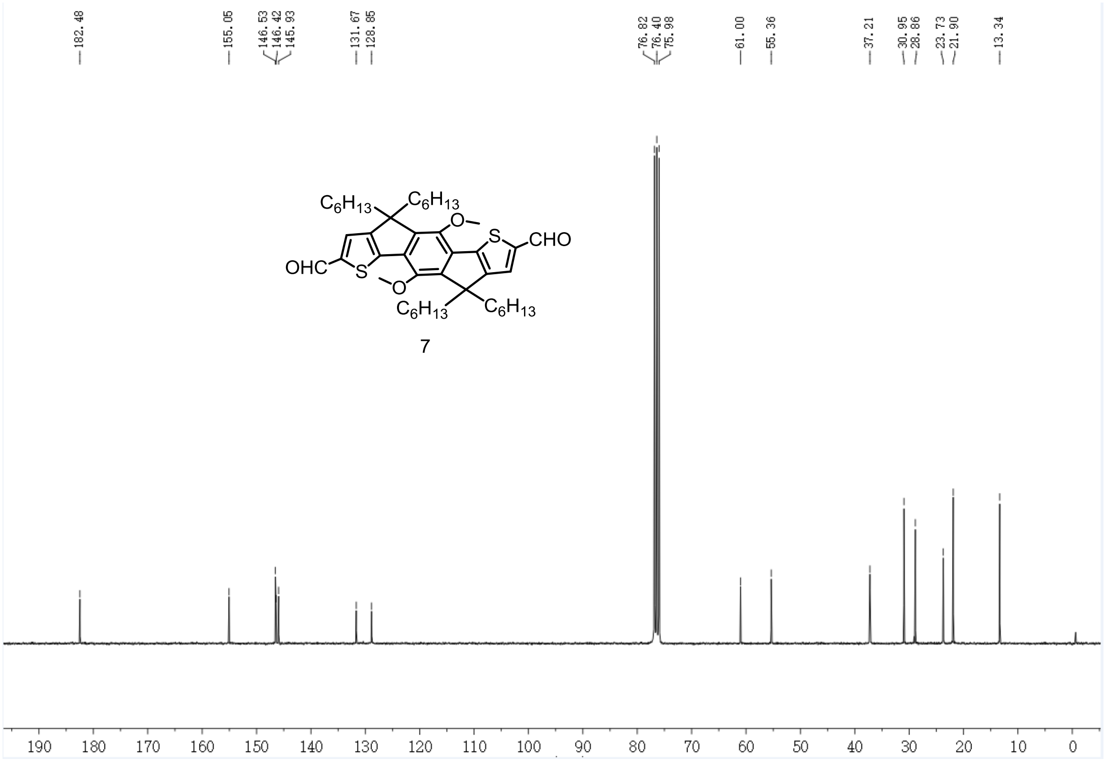

Supplementary Figure 38. ^{13}C NMR spectrum of 7

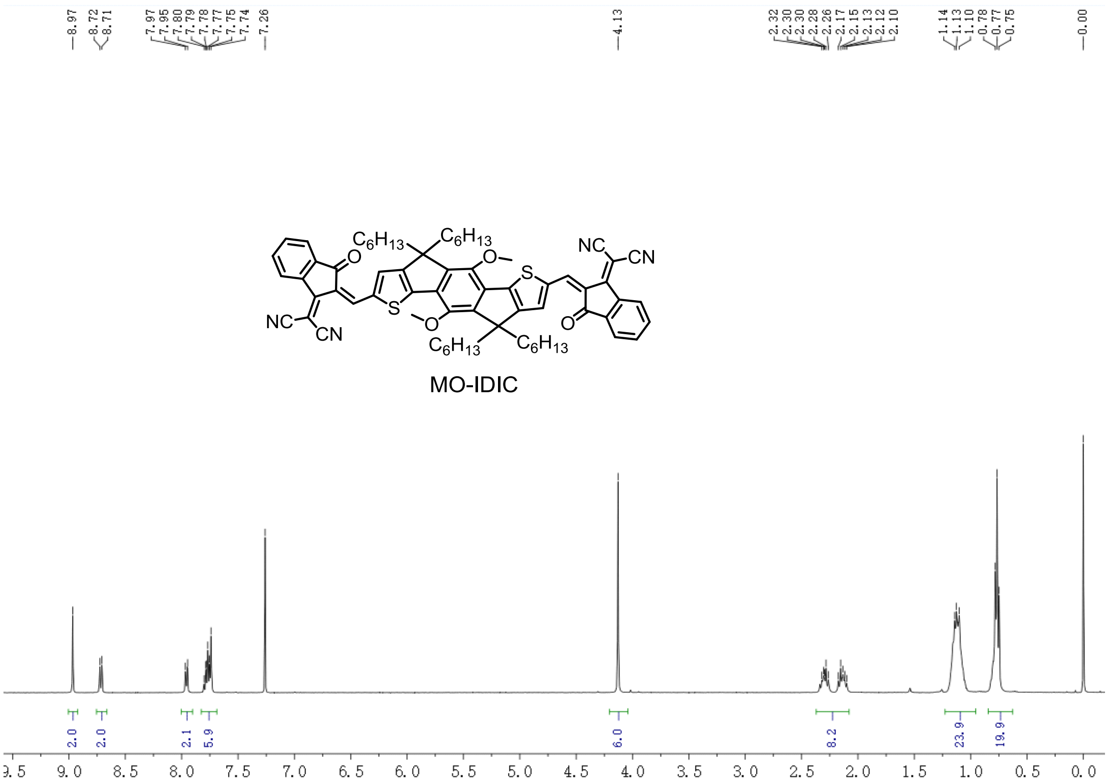

Supplementary Figure 39. ^{1}H NMR spectrum of MO-IDIC

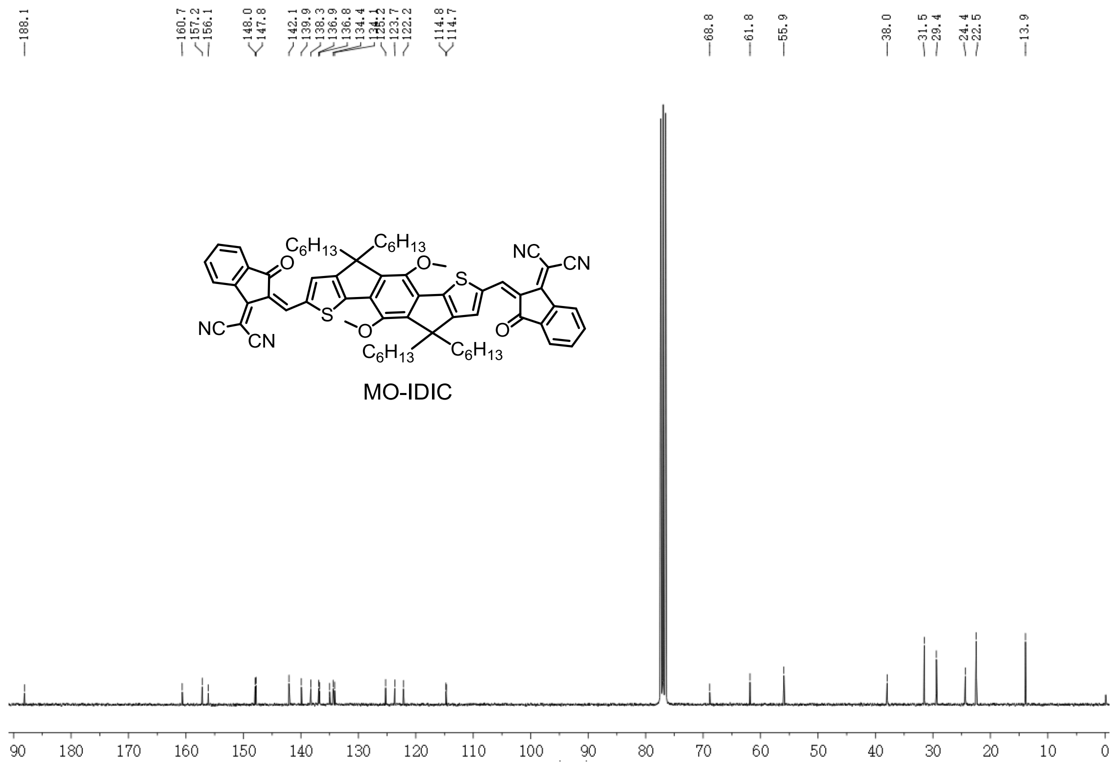

Supplementary Figure 40. ^{13}C NMR spectrum of MO-IDIC

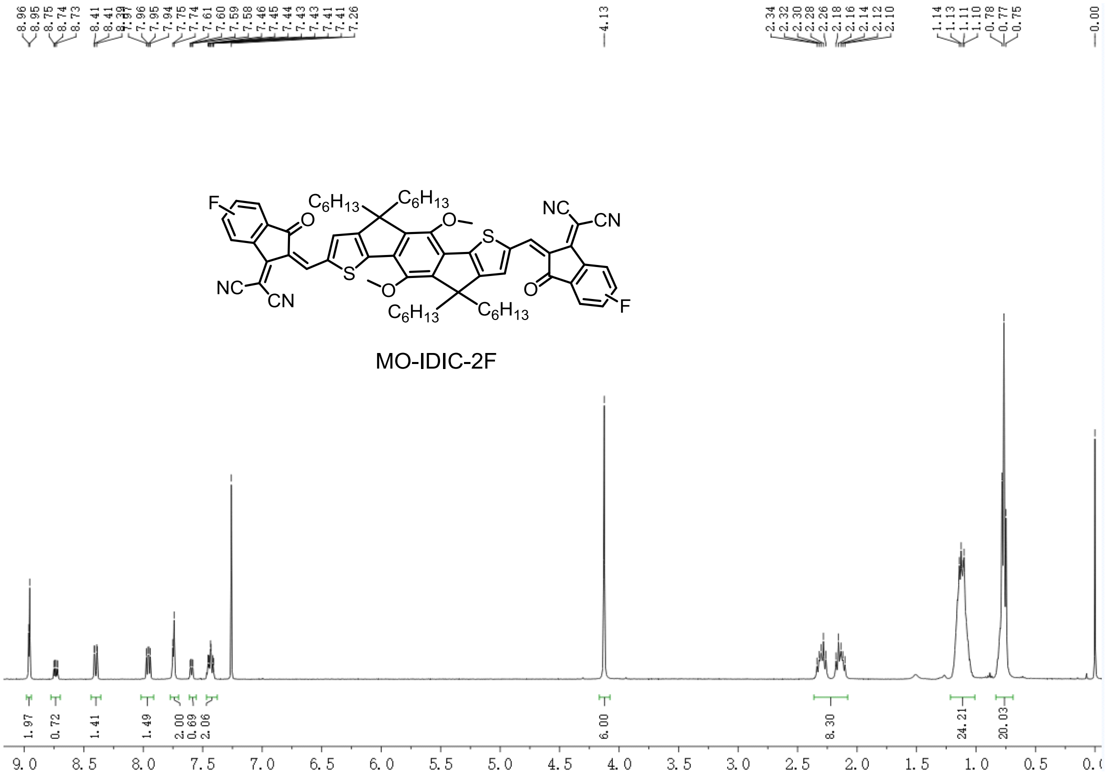

Supplementary Figure 41. ^{1}H NMR spectrum of MO-IDIC-2F

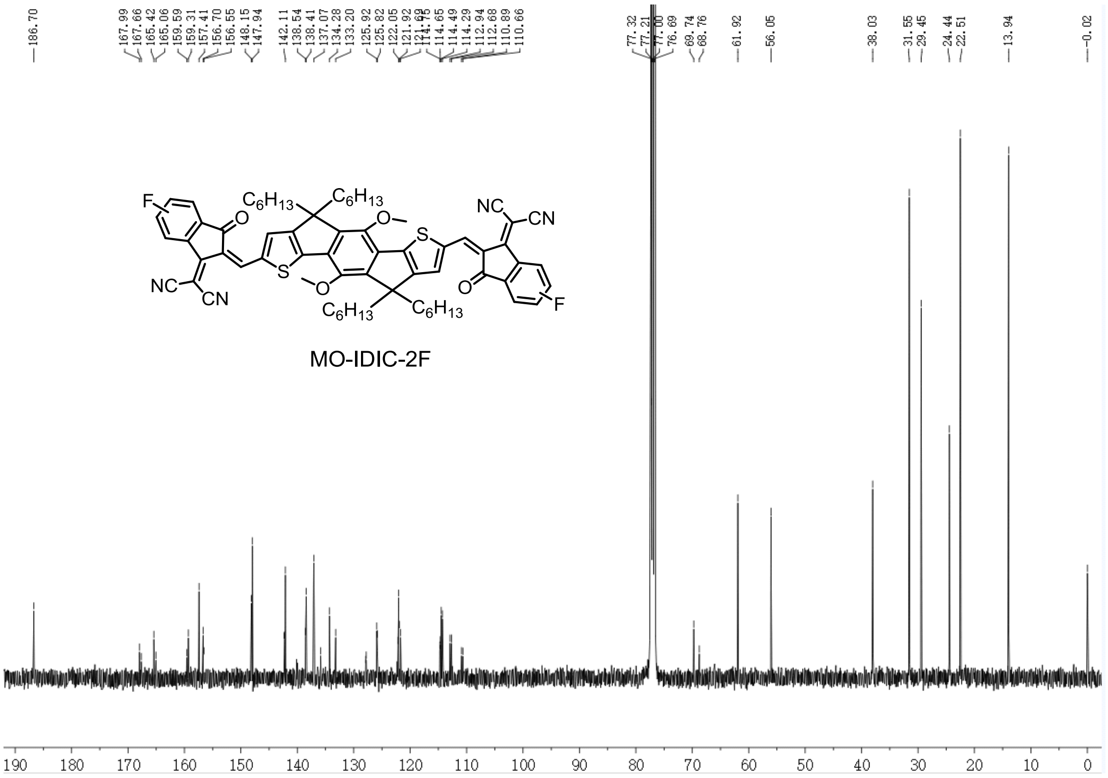

Supplementary Figure 42. ^{13}C NMR spectrum of MO-IDIC-2F

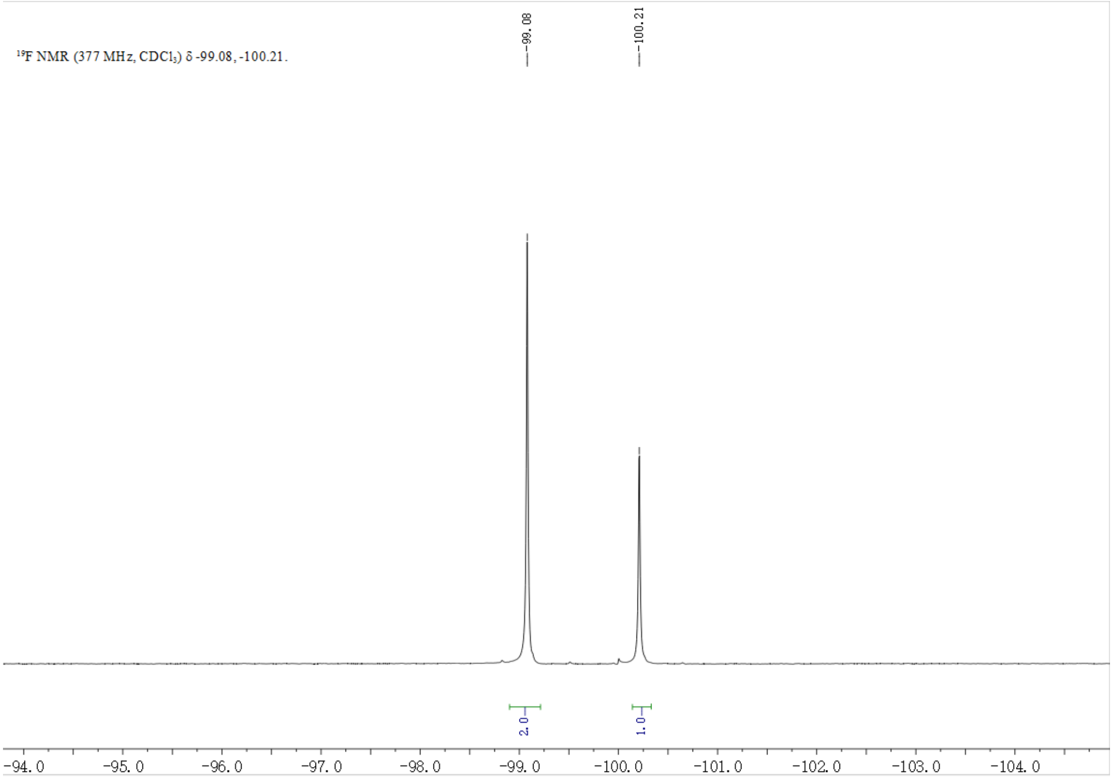

Supplementary Figure 43. ^{19}F NMR spectrum of MO-IDIC-2F

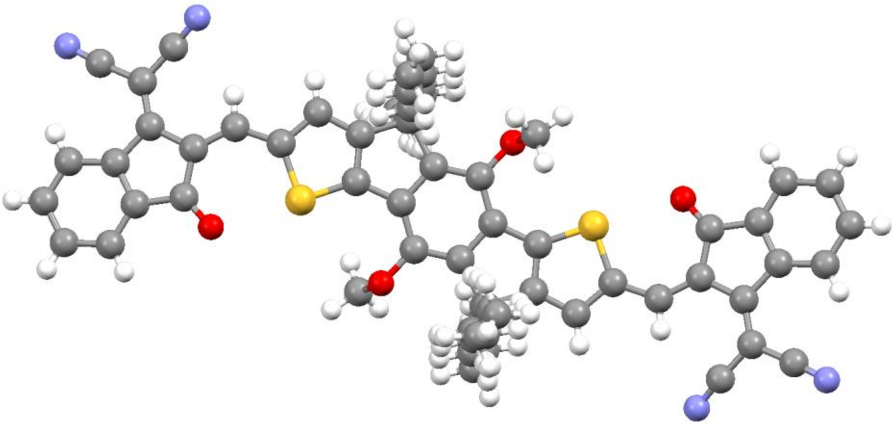

Supplementary Figure 44. Crystal structure of MO-IDIC

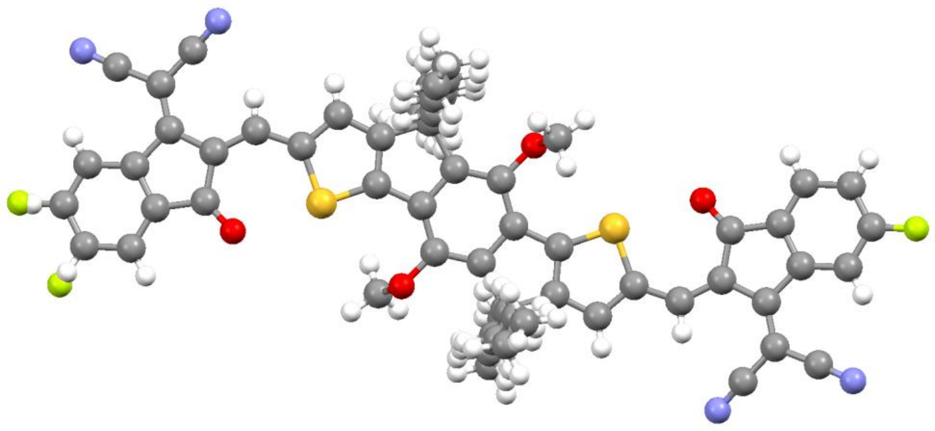

Supplementary Figure 45. Crystal structure of MO-IDIC-2F

Supplementary Tables

Supplementary Table 1. Results of different Friedel-Crafts alkylation ring-closure reaction conditions for the synthesis of Compound **2**.

Entry	Catalist	Equivalent number	Reaction time (h)	Solvent	Temperature (℃)	Target products yield (%)
1	BF_3•OEt_2	1.1eq	12	CH_2Cl_2	rt.	-[a]
2	BF_3•OEt_2	2.2eq	12	CH_2Cl_2	rt.	-[a]
3	BF_3•OEt_2	10eq	12	CH_2Cl_2	rt.	-[b]
4	BF_3•OEt_2	2.2eq	2	CH_2Cl_2	rt.	-[a]
5	H_2SO_4	2.2eq	12	AcOH	80	-[b]
6	HI	2.2eq	12	AcOH	80	-[b]
7	BBr_3	2.2eq	12	Acetonitrile	rt.	-[a]
8	$AlCl_3$	5eq	12	CH_2Cl_2	rt.	15
9	TsOH	2.2eg	12	Toluene	120	-[a]

[a] Mainly a mixture of complex compounds. [b] Mainly the alkene products.

Supplementary Table 2. Physicochemical properties and electronic energy levels of the *n*-OS acceptors.

	λ_{max}[a] (nm)	λ_{edge}[a] (nm)	E_g^{opt}[b] (eV)	E_{HOMO}[c] (eV)	E_{LUMO}[c] (eV)	E_g^{e}[c] (eV)
MO-IDIC	716	776	1.60	-5.69	-3.89	1.80
MO-IDIC-2F	735	800	1.55	-5.80	-3.93	1.87

[a]Absorption of the films. [b] Calculated from the absorption edge of the polymer films: $E_g^{opt} = 1240/\lambda_{edge}$. [c] Calculated according to the equation $E_{LUMO/HOMO} = -e\ (E_{red/ox} + 4.36)$ (eV)

Supplementary Table 3. Charge carrier mobilities of the acceptors and the PSCs based on PTQ10: acceptors.

	μ_h ($cm^2 V^{-1} s^{-1}$)	μ_e ($cm^2 V^{-1} s^{-1}$)	μ_h/μ_e
MO-IDIC	-	8.15×10^{-4}	
MO-IDIC-2F	-	1.01×10^{-3}	
PTQ10:MO-IDIC(as cast)	2.55×10^{-5}	2.55×10^{-4}	0.10
PTQ10:MO-IDIC(annealed)	4.37×10^{-5}	2.97×10^{-4}	0.15
PTQ10:MO-IDIC-2F(as cast)	3.34×10^{-5}	5.28×10^{-4}	0.06
PTQ10:MO-IDIC-2F(annealed)	1.03×10^{-4}	8.09×10^{-4}	0.13

Supplementary Table 4. Photovoltaic performance parameters of the non-fullerene PSCs based on PTQ10: acceptors with different D:A weight ratios and with thermal annealing at 120 ℃ (MO-IDIC) and 110 ℃ (MO-IDIC) for 5 min, under the illumination of AM1.5G, 100 mW cm^{-2}

Acceptors	D:A weight ratio	V_{oc} (V)	J_{sc} (mA cm^{-2})	FF (%)	PCE (%)
MO-IDIC	1.5:1	0.98	16.30	67.35	10.75
	1:1	0.97	17.01	67.52	11.09
	1:1.5	0.96	16.34	68.05	10.67
MO-IDIC-2F	1.5:1	0.91	18.93	72.1	12.32
	1:1	0.90	19.95	73.8	13.28
	1:1.5	0.89	18.52	76.3	12.58

Supplementary Table 5. Photovoltaic performance parameters of the non-fullerene PSCs based on PTQ10: acceptors (1: 1, w/w) at different thermal annealing temperatures for 5 min under the illumination of AM1.5G, 100 mW cm^{-2}.

Acceptors	Annealing Temp. (℃)	V_{oc} (V)	J_{sc} (mA cm^{-2})	FF (%)	PCE (%)
MO-IDIC	As-cast	0.97	15.87	65.8	10.12
	110	0.97	16.05	66.3	10.32
	120	0.97	17.01	67.5	11.09
	130	0.96	15.86	65.4	10.14
	140	0.96	15.59	63.3	9.67
MO-IDIC-2F	As-cast	0.90	18.02	74.8	12.13
	100	0.90	19.21	73.5	12.68
	110	0.90	19.95	73.8	13.28
	120	0.90	19.44	73.8	12.87
	130	0.89	19.2	73.3	12.50

Supplementary Table 6. Photovoltaic Performance Parameters of the PSCs Based on PTQ10:acceptors with the optimized thermal annealing treatment under the illumination of AM1.5G, 100 mW cm^{-2}

Acceptor	V_{oc} (V)	J_{sc} (mA cm^{-2})	FF （%）	PCE (%)
IDIC[a]	0.972	16.61	72.1	11.65
	(0.962±0.004)	(16.61±0.22)	(71.5±0.7)	(11.43±0.10)
IDIC-2F[b]	0.892	18.94	71.7	12.09
	(0.885±0.005)	(18.65±0.38)	(70.7±0.9)	(11.89±0.17)
MO-IDIC-2F[c]	0.906	19.87	74.8	13.46
	(0.896±0.005)	(19.85±0.46)	(73.6±1.5)	(13.10±0.16)

[a] With thermal annealing at 140 ℃ for 5 min; [b] With thermal annealing at 120 ℃ for 5 min. [c]With thermal annealing at 110 ℃ for 5 min.

Supplementary Table 7. Photovoltaic performance parameters of the PSCs with PTQ10 as donor and MO-IDIC-2F as acceptor with different active layer thickness, under the illumination of AM1.5G, 100 mWcm^{-2}.

Acceptor	Active layer thickness (nm)	V_{oc} (V)	J_{sc} (mA/cm^2)	FF (%)	PCE (%)
MO-IDIC-2F	115	0.907	18.07	75.2	12.32
	127	0.905	19.75	74.8	13.40
	150	0.896	19.80	72.4	12.86
	180	0.902	19.36	73.5	12.85
	222	0.896	20.07	72.0	12.94
	250	0.899	19.93	70.4	12.63
	300	0.885	20.66	60.2	11.01

Supplementary Table 8. Survey of calculated chemical synthesis costs for intermediate materials (100g).

IC	Reagents	1,3-indandione	92g	1564	3473.2
		malononitrile	76g	118.6	
		sodium acetate	62g	1.9	
	Solvent	ethanol	1.6L	21.8	
	Purification	Eluent and extraction solvent	78L	1404	
		SiO_2	12.1kg	363	
IC-1	Reagents	4,5-difluorophthalic anhydride	149g	19660	24095
		ethyl acetoacetate	0.2L	25.6	
		trimethylamine	1.1L	44	
		malononitrile	122g	190.3	
		sodium acetate	96g	3	
	Solvent	ethanol	2.5L	34	
		acetic anhydride	2.1L	231	
	Purification	$MgSO_4$	169g	8.5	
		Eluent and extraction solvent	241 L	3173	
		SiO_2	32.2kg	726	
IC-2	Reagents	5-fluor-indan-1,3-dion	171g	6869	12015
		ethyl acetoacetate	0.19L	25	
		sodium acetate	91g	2.8	
		trimethylamine	0.8L	32	
		malononitrile	110g	171.6	
	Solvent	ethanol	2.2L	29.9	
		acetic anhydride	1.5L	165	
	Purification	$MgSO_4$	140g	7	
		Eluent and extraction solvent	214L	3852	
		SiO_2	28.7kg	861	
IC-3	Reagents	4-methylphthalic anhydride	213g	2939	8723.3
		ethyl acetoacetate	0.29L	408	
		sodium acetate	62g	2	
		trimethylamine	0.69L	28	
		malononitrile	75g	117	
	Solvent	ethanol	1.5L	20.4	
		acetic anhydride	1.8L	198	
	Purification	$MgSO_4$	150g	7	
		Eluent and extraction solvent	228L	4104	

		SiO_2	30kg	900	
4,7-bis(5-bromothiophen-2-yl)-5,6 difluoro-2-(2hexyldecyl)-2H benzo[d][1,2,3]triazole	Reagents	4,5-difluorobenzene-1,2-diamine	36g	570.2	10541
		$NaNO_2$	20.5g	0.7	
		potassium tert-butoxide	77g	50.5	
		1-hexyl-decane	118g	2289.7	
		2-bromothiophene	94g	129.72	
		$Pd(TFA)_2$	3.2g	634.9	
		Ag_2O	178g	2937	
	Solvent	DMSO	2.9L	147.9	
		AcOH	0.03L	0.7	
		methanol	0.6L	15	
	Purification	$MgSO_4$	173g	8.7	
		Eluent and extraction solvent	174L	3132	
		SiO_2	20.8kg	624	
1,3-Bis(5-bromothiophen-2-yl)-5,7-bis(2-ethyl hexyl) benzo[1,2-c:4,5-c’] dithiophene-4,8-dione	Reagents	thiophene-3,4-dicarboxylic acid	70g	3118.8	23289
		Br_2	70g	9.1	
		oxalyl chloride	0.12L	66	
		$AlCl_3$	100g	4.5	
		2,5-fibromothiophene	183g	1535.4	
		2-ethylhexyl bromide	703g	844	
		$Pd(PPh_3)_4$	1.6g	80	
		thiophene	55g	55	
		n-BuLi	0.29l	126	
		trimethylchlorotin	0.78L	9555	
		N-bromosuccinimide (NBS)	41g	22	
	Solvent	DMF	0.9L	22	
		AcOH	0.16L	3.4	
		dichloromethane	0.20L	4.8	
		toluene	0.8L	59.2	
	Purification	$MgSO_4$	370g	19	
		Eluent and extraction solvent	357.5L	6435	
		SiO_2	44.3kg	1329	
1,3-bis(5-bromo-4-fl		2-bromothiophen	66g	92	49149
		TMSCl	44g	244	
		1,3-dibromo-5,7-bis(2-ethylhexyl)-4H,8H-benzo[1,2-c:4,5-c']bisthiophene-4,8-dione	65g	3756	

uorothiophen-2-yl)-5,7-bis(2-ethylhexyl)-4H,8H-benzo [1,2-c:4,5-c'] bisthiophene-4,8-dione	Reagents	LDA (2M)	0.24L	225	
		chlorotriisopropylsilane	0.12L	239.4	
		n-BuLi	12.5L	5250	
		N-Fluorobenzenesulfonimide	137g	979.55	
		Br_2	110g	14.3	
		$ZnCl_2$	47.7g	2.194	
		$Pd(PPh_3)_4$	6.3g	315	
		tetrabutylammonium fluoride (TBAF)	123.5g	401.55	
	Solvent	THF	2L	114	
		CH_2Cl_2	19.8L	475.2	
		diethyl ether	3.6L	111.6	
	Purification	$MgSO_4$	780g	40	
		Eluent and extraction solvent	719L	12942	
		SiO_2	798.3kg	23949	
TerT-E-C10	Reagents	2-bromothiophene-3-carboxylic acid	124.8g	8987	20116
		Dicyclohexyl carbodiimide	62.1g	509.2	
		4-dimethylaminopyridine	18.1g	55	
		n-decanol	95.3g	27.6	
		N-Bromosuccinimide	56.6g	40.1	
		thiophene	24.2g	28.1	
		n-BuLi	0.08L	30	
		trimethyltin chloride	0.17L	2109.6	
		$Pd(PPh_3)_4$	10.64g	532.3	
	Solvent	toluene	2.58L	95.5	
		Dichloromethane	1.61L	41.9	
		THF	1.15L	65.5	
	Purification	$MgSO_4$	366.1g	18.9	
		Eluent and extraction solvent	347.7L	6259.3	
		SiO_2	43.9kg	1316.1	

Supplementary Table 9. Survey of calculated chemical synthesis costs for acceptor materials (100g).

Materials			Quantity consumed	Cost (￥)	Total (￥)
IDIC	Reagents	2,5-dibromo-terephthalic acid diethyl ester	156g	2134	26842
		$Pd(PPh_3)_4$	28g	1420	
		2-bromothiophene	0.2L	824	
		$ZnCl_2$	300g	13.5	
		Mg	50g	50	
		potassium hydroxide	450g	15.3	
		hydrochloric acid	3.4L	49	
		oxalyl chloride	170g	718.5	
		$AlCl_3$	210g	24.2	
		hydrazine monohydrate	269g	35.8	
		diethylene glycol	0.18L	8.6	
		DMSO	0.86L	43.9	
		potassium tert-butoxide	108g	103.7	
		1-bromohexane	160g	84.8	
		$POCl_3$	0.27L	112.9	
		DMF	0.80L	20.8	
		pyridine	0.60L	56.4	
		IC	120g	4167.6	
		toluene	1.2L	45	
		THF	6L	342	
		dichloromethane	4.6L	110.4	
		$CHCl_3$	18.4L	632.9	
	Purification	$MgSO_4$	500g	25	
		Eluent and extraction solvent	498L	8964	
		SiO_2	228kg	6840	
		2,5-dibromo-terephthalic acid diethyl ester	156g	2134	
		$Pd(PPh_3)_4$	34.2g	1734	
		2-bromothiophene	0.2L	824	
		$ZnCl_2$	300g	13.5	
		Mg	50g	50	

O-IDTBR	Reagents	potassium hydroxide	450g	15.3	68331
		hydrochloric acid	3.4L	49	
		oxalyl chloride	170g	718.5	
		$AlCl_3$	210g	24.2	
		hydrazine monohydrate	269g	35.8	
		diethylene glycol	0.18L	8.6	
		DMSO	0.86L	43.9	
		potassium tert-butoxide	108g	103.7	
		1-Bromooctane	160g	84.8	
		n-BuLi	0.124L	52	
		2,1,3-benzothiadiazole-4-carboxaldehyde	78.6g	31200	
		3-ethylrhodanine	46.5g	1821	
		trimethyltin chloride	374.5mL	4594	
		tert-butyl alcohol	7.5L	2805	
		toluene	5.5L	206	
		THF	16L	912	
		dichloromethane	4.6L	110.4	
		$CHCl_3$	18.4L	632.9	
	Purification	$MgSO_4$	700g	35	
		Eluent and extraction solvent	698L	12564	
		SiO_2	252kg	7560	
MO-IDIC	Reagents	1,4-Dibromo-2,5-dimethoxybenzene	60g	1440	17387
		2-bromothiophene-3-carboxylic acid ethyl ester	80g	4800	
		n-BuLi (2.5M)	0.78L	328	
		2-isopropoxy-4,4,5,5-tetramethyl-[1,3,2] dioxaborolane	80g	153.6	
		Pd $(OAc)_2$	1.85g	362.6	
		t-Bu_3PHBF_4	4.76g	120.3	
		1-bromohexane	150g	79.5	
		Mg	22g	22	
		amberlyst15	60g	144.5	
		$POCl_3$	0.24L	100.7	
		DMF	0.78L	20.3	
		pyridine	0.32L	30.8	
		IC	103g	3577.2	

	Solvent	THF	5.4L	307.8	
		acetone	3L	54	
		toluene	1.2L	44.4	
		$CHCl_3$	21.2L	729.3	
	Purification	$MgSO_4$	210g	12	
		Eluent and extraction solvent	228L	4180	
		SiO_2	29kg	880	
MO-IDIC -2F	Reagents	1,4-dibromo-2,5-dimethoxybenzene	58.5g	1404	25066
		2-bromothiophene-3-carboxylic acid ethyl ester	79g	4740	
		n-BuLi 2.5M	0.76L	320	
		2-isopropoxy-4,4,5,5-tetramethyl-[1,3,2] dioxaborolane	78g	153	
		Pd $(OAc)_2$	1.85g	362.6	
		$t\text{-}Bu_3PHBF_4$	4.7g	120.3	
		1-bromohexane	150g	79.5	
		Mg	22g	22	
		amberlyst15	60g	144.5	
		$POCl_3$	0.24L	98.23	
		DMF	0.76L	19.76	
		Pyridine	0.32L	30.8	
		IC-2	96g	11534	
	Solvent	THF	3.7L	210.9	
		acetone	2.8L	50.4	
		toluene	1.2L	44.4	
		$CHCl_3$	21L	722.4	
	Purification	$MgSO_4$	205g	12	
		Eluent and extraction solvent	229L	4122	
		SiO_2	29.2kg	876	
	Reagents	2,5-dibromo-terephthalic acid diethyl ester	83g	1135	
		Thieno[3,2-b]thiophene	82g	4429	
		n-BuLi (2.5M)	0.31L	128	
		$ZnCl_2$	78g	3.5	
		$Pd(PPh_3)_4$	10g	500	
		4-hexyl-1-bromobenzene	184g	2708.5	
		$POCl_3$	200	83.6	

ITIC		DMF	600	15.6	24511
		pyridine	0.44L	41.4	
		IC	122g	4238	
	Solvent	THF	5L	285	
		acetic acid	4L	91.2	
		$CHCl_3$	28L	963.2	
	Purification	$MgSO_4$	280g	14	
		Eluent and extraction solvent	452L	8129	
		SiO_2	58.2kg	1746	
ITIM	Reagents	2,5-dibromo-terephthalic acid diethyl ester	83 g	1135	29218
		Thieno[3,2-b]thiophene	82 g	4429	
		n-BuLi (2.5M)	300mL	126	
		$ZnCl_2$	75g	3.4	
		$Pd(PPh_3)_4$	9.6g	480	
		4-hexyl-1-bromobenzene	178g	2620.2	
		$POCl_3$	196mL	81.9	
		DMF	580mL	15.1	
		pyridine	260mL	24.4	
		IC-3	110g	9595.3	
	Solvent	THF	1.6L	91.2	
		acetic acid	3.85L	87.8	
		$CHCl_3$	26L	894.4	
	Purification	$MgSO_4$	271g	13.6	
		Eluent and extraction solvent	446 L	8028	
		SiO_2	53.1kg	1593	
	Reagents	2,5-dibromo-terephthalic acid diethyl ester	71g	971	
		Thieno[3,2-b]thiophene	81g	4375	
		n-BuLi (2.5M)	0.32L	130	
		$ZnCl_2$	82g	4	
		$Pd(PPh_3)_4$	10.5g	525	
		4-hexyl-1-bromobenzene	193g	2840.9	
		$POCl_3$	0.21L	87.8	
		DMF	0.62L	16.1	

ITIC-4F		Pyridine	0.23L	21.6	43435
		IC-1	100g	24095	
	Solvent	THF	1.6L	91.2	
		$CHCl_3$	20L	688	
		acetic acid	4L	91.2	
	Purification	$MgSO_4$	297g	15	
		Eluent and extraction solvent	430L	7740	
		SiO_2	58.1kg	1743	
C8-ITIC	Reagents	LDA (2M)	0.480L	1250	78335
		2,7-dibromo-4,4,9,9-tetraoctyl-4,9-dihydro-s-indaceno[1,2-b:5,6-b']bisthiophene	211g	55438	
		ethyl mercaptoacetate	0.081L	203	
		K_2CO_3	152g	6.992	
		$LiAiH_4$ (1M)	0.76L	2557.1	
		Dess-Martin periodinane	146g	988.4	
		pyridine	0.22L	20.4	
		IC	96g	3334	
	Solvent	THF	27L	1539	
		$CHCl_3$	24L	825.6	
		DMF	11L	286	
	Purification	$MgSO_4$	490g	25	
		Eluent and extraction solvent	542L	9756	
		SiO_2	70.2kg	2106	
NITI	Reagents	sodium methoxide	39g	13.26	73302
		ethyl phenylacetate	0.12L	29.1	
		iodine	100g	119.2	
		potassium hydroxide	130g	4.4	
		hydrochloric acid	0.12L	1.7	
		phosphorus pentachloride	130g	92.8	
		acetic acid	1.3L	29.6	
		zinc dust	375g	28.1	
		n-BuLi (2.5M)	9.8L	4116	
		2-ethylhexyl bromide	0.23L	685.4	
		carbon tetrachloride	67L	3484	
		$CuBr_2$	540g	140	
		2-isopropoxy-4,4,5,5	14g	39	

		tetramethyl-1,3,2-dioxaborolane		
		2-thiophenecarboxylic acid	794g	1191
		quinoline	2.6L	734.6
		bariumpromoted copper chromite	128g	401.2
		$Pd(PPh_3)_4$	12g	600
		NBS	97g	50.2
		$POCl_3$	0.50L	209
		Pyridine	0.23L	21.6
		IC-1	91g	21926
	Solvent	THF	200L	11400
		polyphosphoric acid	18.8L	11768.8
		$CHCl_3$	0.25L	8.6
		benzene	36L	864
		DMF	0.90L	23.4
		$CHCl_3$	12L	412.8
	Purification	$MgSO_4$	516g	26
		Eluent and extraction solvent	695L	12510
		SiO_2	92.4kg	2772

Supplementary Table 10. Survey of calculated chemical synthesis costs for donor materials (100g).

PBDB-T	Reagents	thiophene	75g	75	36721
		n-BuLi	2.33L	979	
		2-ethylhexyl bromide	0.16L	465	
		4,8-diketobenzodithiophene	36g	1800	
		tin dichloride dihydrate	290g	74	
		hydrochloric acid	0.36L	5	
		trimethyltin chloride	0.37L	4532.5	
		1,3-Bis(5-bromothiophen-2-yl)-5,7bis(2ethylhexyl) benzo [1,2-c:4,5-c’] dithiophene-4,8-dione	106g	24771	
		$Pd(PPh_3)_4$	9.3g	465	
	Solvent	toluene	3.7l	136.9	
		THF	5L	331	
	Purification	$MgSO_4$	160g	8	
		Eluent and extraction solvent	85.8L	1544	
		SiO_2	8.5kg	255	
		methanol	15L	255	
		hexanes	15L	510	
		chloroform	15L	516	
	Reagents	3-bromothiophene	298g	405.3	
		LDA (2M)	1.35L	1266	
		sulfur powder	60g	10.6	
		2-ethylhexyl bromide	640g	726.7	
		4,8-diketobenzodithiophene	114g	5700	
		tin dichloride dihydrate	863g	210	
		hydrochloric acid	0.20L	2.8	
		trimethylchlorosilane	0.47L	280.4	
		n-BuLi	0.47L	200	
		N-fluorobenzenesulfonamide	260g	1859	
		trifluoroacetic acid	0.82L	1230	
		trimethyltin chloride	0.54L	6615.6	
		1,3-bis(5-bromothiophen-2-yl)-5,7 bis(2ethylhexy l) benzo[1,2-c:4,5-c’] dithiophene-4,8-dione	79g	18696	

PBDB-T-SF		$Pd(PPh_3)_4$	7.4g	370	63408
	Solvent	toluene	3.5L	129.5	
		THF	19.5L	1111.5	
		chloroform	0.92L	68.8	
	Purification	$MgSO_4$	1.09kg	55	
		Eluent and extraction solvent	1069L	19242	
		SiO_2	131.9kg	3957	
		methanol	15L	255	
		hexanes	15L	510	
		chloroform	15L	516	
PFDBD-T	Reagents	3-bromothiophene	298g	405	79663
		LDA	2.67L	2503	
		sulfur powder	60g	10.6	
		2-ethylhexyl bromide	640g	768	
		4,8-diketobenzodithiophene	47g	2350	
		tin dichloride dihydrate	863g	210	
		hydrochloric acid	0.20L	2.8	
		trimethylchlorosilane	0.41L	244	
		n-BuLi	0.47L	200	
		N-fluorobenzenesulfonamide	270g	1930	
		trifluoroacetic acid	1.9l	2850	
		trimethyltin chloride	0.45L	5450	
		1,3-bis(5-bromo-4-fluorothiophen-2-yl)-5,7 bis(2-ethylhexyl)-4H, 8H-benzo [1,2-c:4,5c']bisthiophene-4,8-dione	78g	38336	
		$Pd(PPh_3)_4$	4g	200	
	Solvent	toluene	6L	222	
		THF	19.5L	1112	
		chloroform	2l	150	
	Purification	$MgSO_4$	1.05kg	55	
		Eluent and extraction solvent	988L	17784	
		SiO_2	118.6kg	3558	
		methanol	15L	255	
		hexanes	15L	510	
		chloroform	15L	516	
		3-bromothiophene	238.4g	324.24	
		LDA (2M)	1.08L	1012.8	

PDTB-EF-T(P2)	Reagents	sulfur powder	48g	8.48	48105
		2-ethylhexyl bromide	512g	581.36	
		4,8-diketobenzodithiophene	91.2g	4560	
		tin dichloride dihydrate	690.4g	168	
		hydrochloric acid	0.16L	2.24	
		trimethylchlorosilane	0.376L	224.32	
		n-BuLi	0.376L	160	
		N-fluorobenzenesulfonamide	208g	1487.2	
		trifluoroacetic acid	0.656L	984	
		trimethyltin chloride	0.432L	5292.48	
		TerT-E-C10	62g	12472	
	Solvent	THF	15.6L	889.2	
		chloroform	0.736L	55.04	
		toluene	3.7L	136.9	
	Purification	$MgSO_4$	0.872kg	44	
		Eluent and extraction solvent	855.2L	15393.6	
		SiO_2	105.52Kg	3165.6	
		methanol	15L	255	
		hexanes	15L	510	
		chloroform	15L	516	
PBDB-T-2Cl	Reagents	3-chlorothiophene	190g	3526.6	46587
		LDA	0.97L	909.6	
		n-BuLi	0.68L	289	
		2-ethylhexyl bromide	0.37L	1075	
		4,8-diketobenzodithiophene	89g	4450	
		tin dichloride dihydrate	604g	154	
		hydrochloric acid	1.28L	17.7	
		trimethyltin chloride	0.55L	6737.5	
		1,3-Bis(5-bromothiophen-2-yl)-5,7bis(2ethylhexyl) benzo [1,2-c:4,5-c’] dithiophene-4,8-dione	101g	23603	
		$Pd(PPh_3)_4$	6.6g	330	
	Solvent	toluene	3.7L	136.9	
		THF	11.36L	752	
	Purification	$MgSO_4$	272g	13.6	
		Eluent and extraction solvent	155.3L	2795	
		SiO_2	17.2kg	516	

		methanol	15L	255	
		hexanes	15L	510	
		chloroform	15L	516	
PBDB-T-2F	Reagents	3-bromothiophene	405g	550.8	77872
		LDA (2M)	3.63L	3398.4	
		2-ethylhexyl bromide	870g	987.9	
		4,8-diketobenzodithiophene	64g	3200	
		tin dichloride dihydrate	1173g	286.2	
		hydrochloric acid	0.27L	3.8	
		trimethylchlorosilane	560g	446.9	
		n-BuLi (2.5M)	0.64L	270	
		N-fluorobenzenesulfonamide	500g	3575	
		trifluoroacetic acid	1.58L	1516.8	
		trimethyltin chloride	0.61L	7423.5	
		1,3-Bis(5-bromothiophen-2-yl)-5,7bis(2ethylhexyl) benzo [1,2-c:4,5-c’] dithiophene-4,8-dione	105g	24538	
		$Pd(PPh_3)_4$	6.6g	330	
	Solvent	toluene	3.7L	136.9	
		THF	26L	1482	
		chloroform	2.8L	96.3	
	Purification	$MgSO_4$	1.33kg	67	
		Eluent and extraction solvent	1303L	23454	
		SiO_2	160.9kg	4827	
		methanol	15L	255	
		hexanes	15L	510	
		chloroform	15L	516	
	Reagents	3-bromothiophene	405g	550.8	
		LDA (2M)	3.63L	3398.4	
		2-ethylhexyl bromide	870g	987.9	
		4,8-diketobenzodithiophene	64g	3200	
		tin dichloride dihydrate	1173g	286.2	
		hydrochloric acid	0.27L	3.8	
		trimethylchlorosilane	560g	446.9	
		n-BuLi (2.5M)	0.64L	270	
		N-fluorobenzenesulfonamide	500g	3575	
		trifluoroacetic acid	1.58L	1516.8	

PBTA-TF		trimethyltin chloride	0.61L	7423.5	63949
		4,7-bis(5-bromothiophen-2-yl)-5,6-difluoro-2-(2-hexyldecyl)-2H-benzo[d][1,2,3]triazole	98g	10330	
		$Pd_2(dba)_3$	3.9g	421.2	
		$P(o\text{-}tol)_3$	5.1g	40.2	
	Solvent	toluene	7.9L	292.3	
		THF	26L	1482	
		chloroform	2.8L	96.3	
	Purification	$MgSO_4$	1.33kg	67	
		Eluent and extraction solvent	1303L	23454	
		SiO_2	160.9kg	4827	
		methanol	15L	255	
		hexanes	15L	510	
		chloroform	15L	516	
PBDTS-TDZ	Reagents	3-hexylthiophene	452g	7440	43645
		copper (I) cyanide	367g	470	
		quinoline	3.14L	1243	
		potassium hydroxide	211g	7.2	
		thionyl chloride	0.34l	573	
		triethylamine	0.17L	6.8	
		hydrazine monohydrate	0.02L	2.9	
		Lawesson's reagent	121g	580	
		NBS	65.4g	34	
		thiophene	61g	61	
		n-BuLi	0.67L	283	
		2-butyloctylbromine	180g	410	
		4,8-Diketobenzodithiophene	34g	1700	
		tin dichloride dihydrate	275g	67.3	
		hydrochloric acid	0.50L	7	
		trimethyltin chloride	0.20L	2487	
		sulfur powder	24g	10	
		$Pd_2(dba)_3$	1.9g	216	
		$P(o\text{-}tol)_3$	2.5g	20	
	Solvent	ethylene glycol	2.3l	1532	
		N-methyl pyrrolidone	1.42L	586	
		toluene	4.1L	91	

		DMF	0.9L	26	
		THF	5.37L	310	
	Purification	$MgSO_4$	1290g	65	
		Eluent and extraction solvent	1112L	20016	
		SiO_2	138kg	4140	
		methanol	15L	255	
		hexanes	15L	510	
		chloroform	15L	516	
PTQ10	Reagents	3,6-dibromo-4,5-difluorobenzene-1,2-diamine	74g	11100	25928
		glyoxylic acid	18g	48	
		potassium tert-butanolate	33g	21.6	
		1-bromo-2-hexyldecane	73g	1416.5	
		thiophene	89g	102.4	
		n-BuLi	0.26L	110	
		trimethyltin chloride	0.63L	7717.5	
		$Pd(PPh_3)_4$	8.7g	435	
	Solvent	acetic acid	2.4L	54.7	
		methanol	2.5L	62.5	
		THF	4.2L	239.4	
		toluene	11L	407	
	Purification	$MgSO_4$	160g	8	
		Eluent and extraction solvent	118L	2124	
		SiO_2	26.7kg	801	
		methanol	15l	255	
		hexanes	15l	510	
		chloroform	15l	516	
P3HT	Reagents	3-hexylthiophene	188g	3095.2	7006.5
		N-bromosuccinimide	265g	137.3	
		methylmagnesium bromide (1M)	0.62L	1091.2	
		$Ni(dppp)Cl_2$	3.4g	28.6	
	Solvent	THF	22.6L	1288.2	
		hexane	2.5L	85	
	Purification	methanol	15L	255	
		hexanes	15L	510	
		chloroform	15L	516	

Supplementary Table 11. Survey of the synthetic steps and synthesis costs for donor and acceptor materials

Compound	Total Step	C_g (¥ per g)	Reference
IDIC	9	268.4	1,2
MO-IDIC	6	173.8	This work
MO-IDIC-2F	7	250.6	This work
ITIC	6	245.1	3-7
ITIM	7	292.2	3, 8
ITIC-4F	7	434.4	9-11
C8-ITIC	13	783.3	2, 12
NITI	12	733.0	13-16
O-IDTBR	10	683.3	17
PBDB-T	10	367.2	18-19
PBDB-T-SF	13	634.1	9, 20
PFDBD-T	17	796.6	12, 20
PBTA-TF	11	639.5	20-21
PBDTS-TDZ	10	436.5	18, 22
PDTB-EF-T(P2)	10	481.1	19,23
PBDB-T-2Cl	10	465.9	24
PBDB-T-2F	11	778.7	21,24,25
PTQ10	3	259.3	26
P3HT	2	70.1	27

Supplementary Discussion:

Transient absorption studies

To investigate the mechanism underlying the devices based on the two acceptors, we performed fs-resolved transient absorption (TA) spectroscopy measurements with probing light wavelength from 450 nm to 1400 nm. Supplementary Figure 6a displays typical TA spectra recorded from the neat films of donor and acceptors at time delay of 1 ps, showing the main features of ground-state bleaching (GSB) and excited-state absorption (ESA). GSB signals in both films of donor and acceptors appear in the spectral ranges close to their major absorption bands. ESA induced by polaron or exciton appears with a broad band feature in the infrared range of 800-1400 nm in the PTQ10 film. ESA in MO-IDIC-2F and MO-IDIC films show a sharp peak at 875 nm and 850 nm respectively. We focus on the carrier dynamics with optical pump at 720 nm. Supplementary Figure 6b shows TA spectra in the annealed film of PTQ10/MO-IDIC-2F recorded at different delay time. Following the decay of GSB and ESA of MO-IDIC-2F, GSB of PTQ10 and ESA centered at 960 nm simultaneously build up. The excitation photon energy (at 720 nm) is much smaller than that required for exciton absorption of PTQ10, suggesting that the initial kinetics is enabled by hole transfer process from photoexcited MO-IDIC-2F to PTQ10. Similar analysis can be applied to the TA data of PTQ10/MO-IDIC recorded in Supplementary Fig. 6c. To figure out the difference in these two blend films, we compare the kinetics recorded at the GSB of PTQ10 (Supplementary Fig. 6d) and PA at 960 nm (Supplementary Fig. 6e). From the kinetic curves in Supplementary Fig. 5d, the initial process of hole transfer is almost same in the blend films of PTQ10/MO-IDIC-2F and PTQ10/MO-IDIC. This can also be examined by similar difference between the GSB signal of acceptor in the neat films and its blend film in two systems (Supplementary Fig. 7). Interestingly, the recombination dynamics exhibits a distinct difference in the two blends. The signal decays much slower in the blend film of PTQ10/MO-IDIC-2F than that in PTQ10/MO-IDIC after hole transfer. Same result is also examined in the decay curves probed at 960 nm. In this temporal scale, the recombination is mainly contributed by the germinate recombination. And

the suppressed germinate recombination in PTQ10/MO-IDIC-2F after hole transfer is likely to be responsible for the improved IPCE in the long-wavelength range. The results agree very well with the better photovoltaic performance of the PTQ10/MO-IDIC-2F-based devices.

Supplementary References

1. Lin, Y. et al. A Facile Planar Fused-Ring Electron Acceptor for As-Cast Polymer Solar Cells with 8.71% Efficiency. *J. Am. Chem. Soc.* **138,** 2973-2976 (2016).
2. Cai, L. et al. 4,9-Dihydro-4,4,9,9-tetrahexyl-s-indaceno[1,2-b:5,6-b']dithiophene as a pi-spacer of donor-pi-acceptor dye and its photovoltaic performance with liquid and solid-state dye-sensitized solar cells. *Org. Lett.* **16,** 106-109 (2014).
3. Wong, K.-T. et al. Syntheses and Structures of Novel Heteroarene-Fused Coplanar π-Conjugated Chromophores. *Org. Lett.* **8**, 5033-5036 (2006).
4. Chen, Y. C. et al. Low-Bandgap Conjugated Polymers for High Efficient Photovoltaic Applications. *Chem. Commun.* **46**, 6503-6505 (2010).
5. Buckle, D. et al. Antiallergic Activity of 2-Nitroindan- 1,3-diones. *J. Med. Chem.* **16**, 1334-1339 (1973).
6. Lin, Y. et al. An electron acceptor challenging fullerenes for efficient polymer solar cells. *Adv. Mater.* **27,** 1170-1174 (2015).
7. Xu, Y. X. et al. Improved charge transport and absorption coefficient in indacenodithieno[3,2-b]thiophene-based ladder-type polymer leading to highly efficient polymer solar cells. *Adv. Mater.* **24,** 6356-6361 (2012).
8. Li, S. et al. Energy-Level Modulation of Small-Molecule Electron Acceptors to Achieve over 12% Efficiency in Polymer Solar Cells. *Adv. Mater.* **28,** 9423-9429 (2016).
9. Zhao, W. et al. Molecular Optimization Enables over 13% Efficiency in Organic Solar Cells. *J. Am. Chem. Soc.* **139,** 7148-7151 (2017).
10. Dai, S. et al. Fused Nonacyclic Electron Acceptors for Efficient Polymer Solar Cells. *J. Am. Chem. Soc.* **139,** 1336-1343 (2017).
11. Yao, H. et al. Design, Synthesis, and Photovoltaic Characterization of a Small Molecular Acceptor with an Ultra-Narrow Band Gap. *Angew.Chem. Int. Ed.* **56**, 3045-3049 (2017).
12. Fei, Z. et al. An Alkylated Indacenodithieno[3,2-b]thiophene-Based Nonfullerene Acceptor with High Crystallinity Exhibiting Single Junction Solar Cell Efficiencies Greater than 13% with Low Voltage Losses. *Adv. Mater.* **30**, *1705209* (2018).
13. Pomerantz, M.; Gu, X. & Zhang, S. Poly(2-decylthieno[3,4-b]thiophene-4,6-diyl). A New Low Band Gap Conducting Polymer. Macromolecules 34, 1817-1822 (2001).
14. Xu, S. J. et al. A Twisted Thieno[3,4-b]thiophene-Based Electron Acceptor Featuring a 14-pi-Electron Indenoindene Core for High-Performance Organic Photovoltaics. *Adv. Mater.* **29,** 1704510 (2017).
15. Zhu, X. et al. New sensitizers for dye-sensitized solar cells featuring a carbon-bridged phenylenevinylene. *Chem. Commun (Camb).* **49,** 582-584 (2013).
16. Song, S. et al. Stabilized Polymers with Novel Indenoindene Backbone against Photodegradation for LEDs and Solar Cells. *Macromolecules* **41**, 7296-7305 (2008).
17. Holliday, S. et al. High-efficiency and air-stable P3HT-based polymer solar cells with a new non-fullerene acceptor. *Nat. Commun* **7,** 11585 (2016).
18. Qian, D. et al. Design, Application, and Morphology Study of a New Photovoltaic Polymer with Strong Aggregation in Solution State. *Macromolecules* **45,** 9611-9617 (2012).
19. Cui, C., Wong, W.-Y. & Li, Y. Improvement of open-circuit voltage and photovoltaic

properties of 2D-conjugated polymers by alkylthio substitution. *Energy Environ. Sci.* **7,** 2276-2284 (2014).

20. Zhang, G. et al. Fluorinated and Alkylthiolated Polymeric Donors Enable both Efficient Fullerene and Nonfullerene Polymer Solar Cells. *Adv. Funct. Mater.* **28,** 1706404 (2018).
21. Zhao, W. et al. Environmentally Friendly Solvent-Processed Organic Solar Cells that are Highly Efficient and Adaptable for the Blade-Coating Method. *Adv. Mater.* **30,** 1704837 (2018).
22. Xu, X. et al. Realizing Over 13% Efficiency in Green-Solvent-Processed Nonfullerene Organic Solar Cells Enabled by 1,3,4-Thiadiazole-Based Wide-Bandgap Copolymers. *Adv. Mater.* **30,** 1703973 (2018).
23. Li, S. et al. A Wide Band-Gap Polymer with a Deep HOMO Level Enables 14.2% Efficiency in Polymer Solar Cells. *J. Am. Chem. Soc.* **140,** 7159-7167 (2018).
24. Zhang, S., Qin, Y., Zhu, J. & Hou, J. Over 14% Efficiency in Polymer Solar Cells Enabled by a Chlorinated Polymer Donor. *Adv. Mater.* **30**, 1800868 (2018).
25. Li, W. et al. A High-Efficiency Organic Solar Cell Enabled by the Strong Intramolecular Electron Push–Pull Effect of the Nonfullerene Acceptor. *Adv. Mater.* **30**, 1707170 (2018).
26. Sun, C. et al. A low cost and high performance polymer donor material for polymer solar cells. *Nat. commun.* **9,** 743 (2018).
27. Loewe, R. S., Khersonsky, S. M & McCullough R. D. A Simple Method to Prepare Head-to-Tail Coupled, Regioregular Poly(3-alkylthiophenes) Using Grignard Metathesis. *Adv. Mater.* **11**, 250-252 (1999).
